# A novel therapeutic strategy of multimodal nanoconjugates for state-of-the-art brain tumor phototherapy

**DOI:** 10.1186/s12951-021-01220-9

**Published:** 2022-01-04

**Authors:** Hyung Shik Kim, Minwook Seo, Tae-Eun Park, Dong Yun Lee

**Affiliations:** 1 Department of Bioengineering, College of Engineering, and BK FOUR Biopharmaceutical Innovation Leader for Education and Research Group, Hanyang University, 222 Wangsimni-ro, Seongdong-gu, Seoul, 04763 Republic of Korea; 2 Department of Biomedical Engineering, College of Information-Bio Convergence Engineering, Ulsan National Institute of Science and Technology (UNIST), Ulsan, 44919 Republic of Korea; 3 Institute of Nano Science and Technology (INST), Hanyang University, Seoul, 04763 Republic of Korea; 4 Elixir Pharmatech Inc., Seoul, 07463 Republic of Korea

**Keywords:** Generation of metal-enhanced reactive oxygen species (MERos), Photodynamic photothermal combination therapy (PDT + PTT), Glioblastoma multiforme (GBM), Gold nanoparticles (AuNPs), Oral absorbable GBM targeting

## Abstract

**Background:**

The outcome of phototherapy, including photothermal therapy (PTT) and photodynamic therapy (PDT) for glioblastoma multiforme (GBM), is disappointing due to insufficient photoconversion efficiency and low targeting rate. The development of phototherapeutic agents that target GBM and generate high heat and potent ROS is important to overcome the weak anti-tumor effect.

**Results:**

In this study, nanoconjugates composed of gold nanoparticles (AuNPs) and photosensitizers (PSs) were prepared by disulfide conjugation between Chlorin e6 (Ce6) and glutathione coated-AuNP. The maximum heat dissipation of the nanoconjugate was 64.5 ± 4.5 °C. Moreover, the proximate conjugation of Ce6 on the AuNP surface resulted in plasmonic crossover between Ce6 and AuNP. This improves the intrinsic ROS generating capability of Ce6 by 1.6-fold compared to that of unmodified-Ce6. This process is called generation of metal-enhanced reactive oxygen species (MERos). PEGylated-lactoferrin (Lf-PEG) was incorporated onto the AuNP surface for both oral absorption and GBM targeting of the nanoconjugate (denoted as Ce6-AuNP-Lf). In this study, we explored the mechanism by which Ce6-AuNP-Lf interacts with LfR at the intestinal and blood brain barrier (BBB) and penetrates these barriers with high efficiency. In the orthotopic GBM mice model, the oral bioavailability and GBM targeting amount of Ce6-AuNP-Lf significantly improved to 7.3 ± 1.2% and 11.8 ± 2.1 μg/kg, respectively. The order of laser irradiation, such as applying PDT first and then PTT, was significant for the treatment outcome due to the plasmonic advantages provided by AuNPs to enhance ROS generation capability. As a result, GBM-phototherapy after oral administration of Ce6-AuNP-Lf exhibited an outstanding anti-tumor effect due to GBM targeting and enhanced photoconversion efficiency.

**Conclusions:**

The designed nanoconjugates greatly improved ROS generation by plasmonic crossover between AuNPs and Ce6, enabling sufficient PDT for GBM as well as PTT. In addition, efficient GBM targeting through oral administration was possible by conjugating Lf to the nanoconjugate. These results suggest that Ce6-AuNP-Lf is a potent GBM phototherapeutic nanoconjugate that can be orally administered.

**Graphical Abstract:**

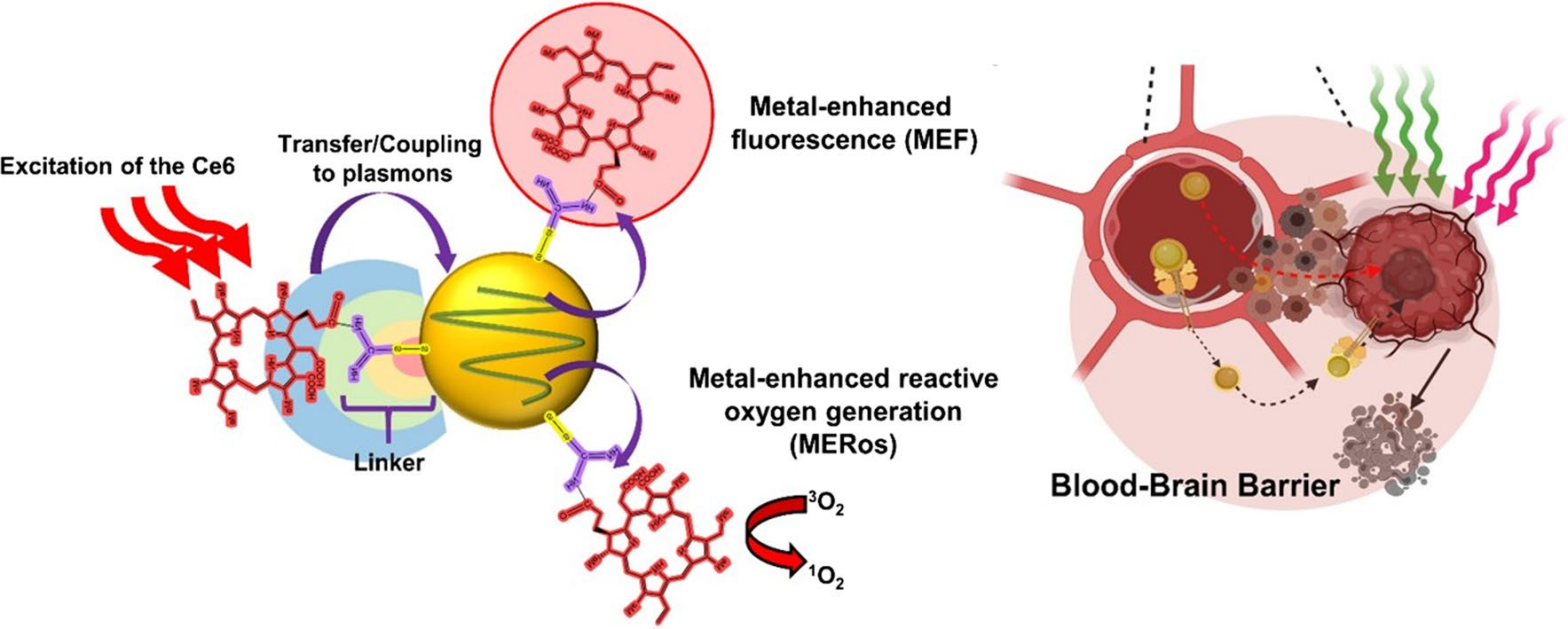

**Supplementary Information:**

The online version contains supplementary material available at 10.1186/s12951-021-01220-9.

## Background

Glioblastoma multiforme (GBM) is the primary grade IV brain malignancy and carries a fatal prognosis. The current standard treatment for GBM involves multimodal therapies including surgical resection, chemotherapy, and radiotherapy. Despite these treatments, the survival rate for patients with GBM is less than 1 year [1, 2]. From a clinical point of view, effective treatment of GBM is limited by several challenges. First, the anatomical complexity and size of the tumor influence the extent of resection. This is because a balance must be achieved between maximum removal of malignant tissue and minimum surgical risk [3]. Even with safe operative resection, residual infiltrating GBM cells that cannot be detected by imaging techniques can remain in the tumor periphery, leading to disease progression and death [4]. Secondly, GBM is characterized by high heterogeneity between intra-tumor and inter-tumor regions at cellular and histological level. This peculiarity of GBM containing tissues induces different responses to therapeutic agents, leading to failure of targeted therapy [5, 6]. Lastly, the central nervous system (CNS) has a distinct microenvironment that is protected by the blood brain barrier (BBB), restricting systemically delivered drugs from accessing the brain. This reduces the treatment options available for GBM. Therefore, development of more sophisticated and powerful treatments is one of the most pressing challenges.

Photothermal therapy (PTT) is based on local application of high temperatures to tissues to induce irreversible cellular damage at the target site and is considered a promising strategy for cancer treatments [7]. It is a non-invasive treatment that involves local irradiation of the tumor using an external near-infrared (NIR) laser. Upon laser irradiation, the photothermal agent harvests light energy, converts it, and releases it as heat [8, 9]. The subsequent temperature increase causes protein denaturation, cellular membrane disruption, enzyme dysfunction, and mitochondrial corruption, leading to tumor cell death and coagulative necrosis [10]. The advantage of PTT as a cancer treatment is that cancerous tissue is more sensitive to heat than is normal tissue, allowing maximum anti-tumor effects while limiting damage to surrounding healthy tissue. This tumor is probably due to the acidic interstitial environment, reduced heat dissipation capacity, and increased metabolic stress [11]. PTT is regarding as a paradigm shifting strategy for non-chemical treatment of GBM using heat to destroy tumors. The method possesses a number of advantages that circumvent the limitations of GBM heterogeneity, drug resistance mechanisms, and adverse effects on surrounding healthy tissues [7]. However, there are challenges to developing nanoparticles that enable effective PTT on GBM. Upon systemic administration, these nanoparticles must overcome the BBB and reach the GBM site at therapeutic concentrations, and the NIR irradiation must cross multiple barriers (skull, scalp, and normal brain tissue) to reach the tumor site without adverse effects.

Photodynamic therapy (PDT) also involves application of light at an appropriate wavelength to activate a photosensitizer (PS). Upon light irradiation, PS initiates relaxation of its electronically excited state through intersystem crossing that leads to production of reactive oxygen species (ROS) [12, 13]. Generation of ROS by activated PS induces a series of physiological responses resulting in cell death. Therefore, PDT is considered a promising cancer therapeutic technique due to its minimally invasive and spatiotemporal properties [12, 14]. Nevertheless, PDT for cancer therapy suffers insufficient tumor accumulation and limited transmittance of external irradiation light, resulting in deficient ROS production. Chlorin e6 (Ce6) is the most widely used porphyrin analog PS in PDT and is a second generation PS with high potency and low dark toxicity [15, 16]. However, application of Ce6 to tumor treatment is limited due to its low water solubility, which prevents its accumulation in tumors at therapeutic levels and cannot lead to sufficient PDT results. The use of 5—40 nm sized AuNPs as Ce6 delivery carriers can address the low water solubility and increase tumor accumulation [17]. Moreover, the surface plasmon resonance (SPR) effect of AuNPs both allows them to be utilized as a PTT agent [18] and amplifies the Raman signal of Ce6 immobilized close to the surface [19–21]. Therefore, a synergistic effect of phototherapy can be anticipated through plasmonic amplification from AuNPs to Ce6, which is directly related to photoconversion efficiency [22].

**Fig. 1:**
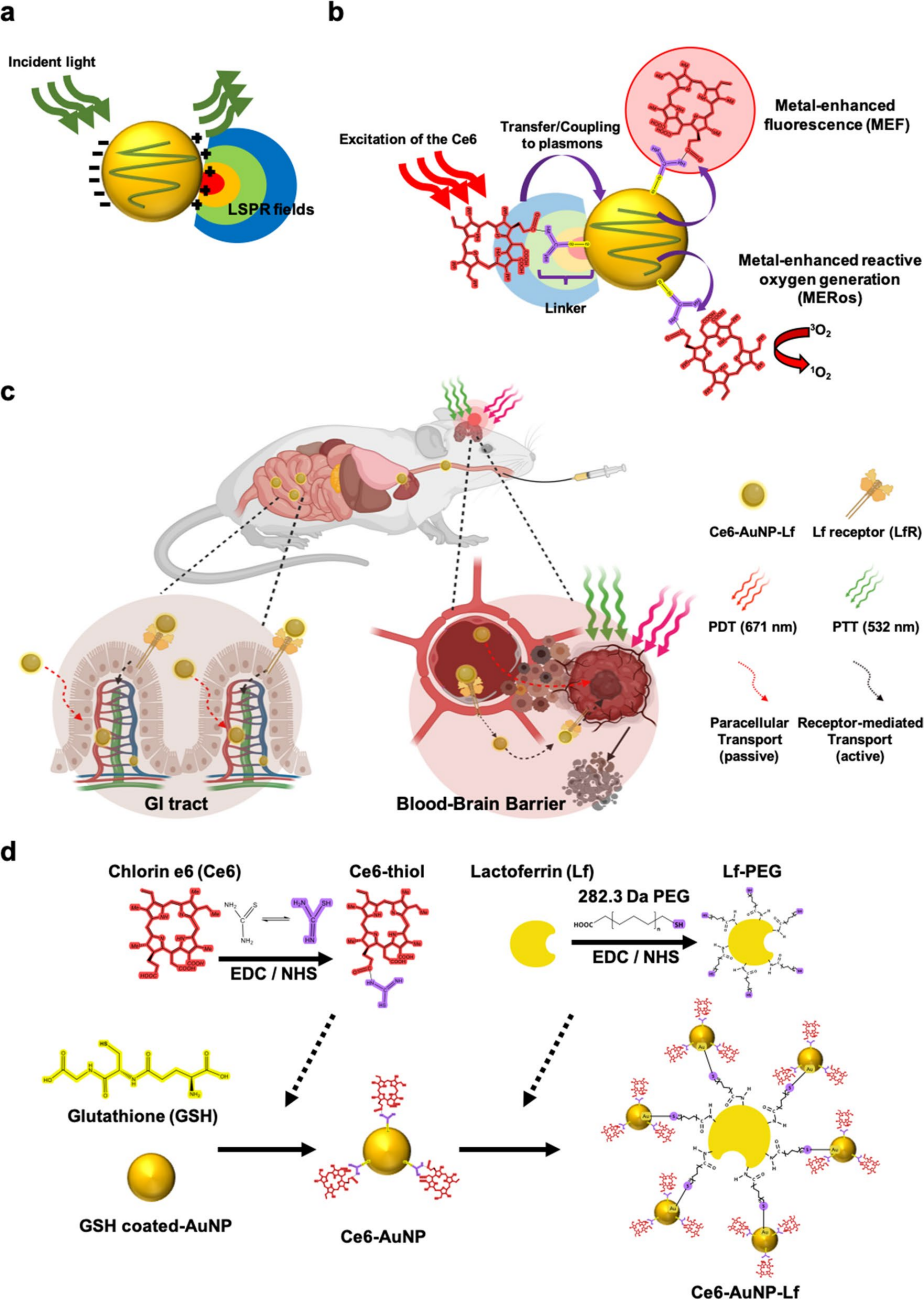
Synthesis of Ce6-AuNP-Lf capable of strong PDT by MERos and the mechanism of GBM treatment. **a** Schematic illustration of AuNP localized surface plasmon resonance (LSPR). **b** Mechanism of metal-enhanced fluorescence (MEF) and metal-enhanced reactive oxygen generation (MERos) by plasmon coupling between AuNP and the conjugated-photosensitizer (PS). **c** Oral absorption and GBM targeting through LfR-mediated pathways of the GI tract, BBB barrier, and GBM cells. Thereafter, the accumulated Ce6-AuNP-Lf in GBM induces apoptosis and necrosis through PTT and PDT, respectively. **d** Synthetic procedure of Ce6-AuNP-Lf by thiolation of Chlorin e6 (Ce6) and PEGylation of Lactoferrin (Lf). The illustration was created with BioRender.com

Metal enhanced fluorescence (MEF) is a phenomenon in which strong surface plasmons of the metal enhances the fluorescence and Raman signals of molecules adsorbed close to the metal surface (Fig. 1a) [23]. In a study examining AuNP carrying PSs [24, 25], fluorescence of conjugated Ce6 on the AuNPs surface was enhanced threefold, and ROS production was increased 1.4-fold. Therefore, synergistically increasing MEF and metal enhanced ROS generation (MERos) can be maximized through conjugation between AuNPs and Ce6 (Fig. 1b). Consequently, this strategy can be used as a tumor treatment that simultaneously applies high-efficiency PDT and PTT [26].

Oral administration is the preferred mode of drug delivery, primarily due to its simplicity and patient convenience. In this regard, lactoferrin (Lf) is generally delivered orally and is absorbed by interactions with the lactoferrin receptor (LfR) expressed in intestinal endothelial cells, BBB, and GBM. Therefore, Lf was conjugated to the AuNPs surface to improve the low oral bioavailability and GBM targeting of AuNPs as described in our previous study [27].

Herein, we developed Ce6-AuNP-Lf that conjugated glutathione-coated AuNP (GSH-AuNP) with the photosensitizer Ce6 and with Lf via a polyethylene glycol (PEG) linker. By proximately conjugating AuNP and Ce6, potent PDT and PTT were available by MERos in GBM. Furthermore, in the GI tract and BBB, both passive and receptor-mediated transport of Ce6-AuNP-Lf was achieved by nano-size and LfR-mediated transcytosis, respectively. The highly efficient targeting of Ce6-AuNP-Lf to GBM by oral administration resulted in a concentration of 11.8 ± 2.1 μg/kg. Afterward, GBM was effectively eradicated with dual treatments including PTT and PDT, which were improved by MERos of Ce6-AuNP-Lf (Fig. 1c).

## Results and discussion

### Preparation and characterization of Ce6-AuNP-Lf nanoconjugate

**Fig. 2:**
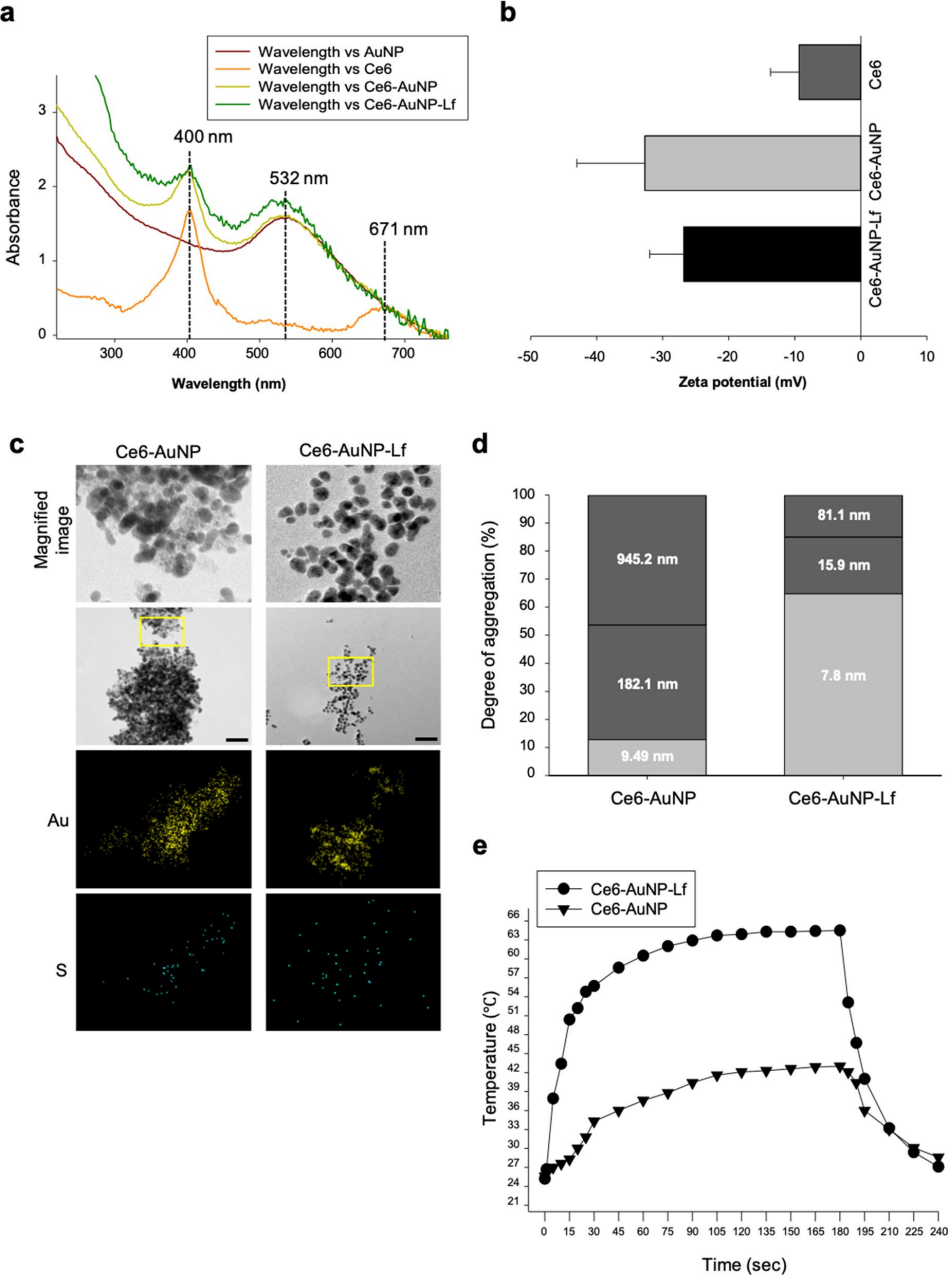
Characterization of Ce6-AuNP-Lf.** a** UV–vis absorbance spectra of AuNP, Ce6, Ce6-AuNP and Ce6-AuNP-Lf having specific peaks at 400, 532 and 671 nm wavelengths. **b** Surface charge of Ce6, Ce6-AuNP and Ce6-AuNP-Lf. Data were expressed as mean ± SEM (n = 3). **c** TEM images and energy-dispersive X-ray spectroscopy (EDX) result of Ce6-AuNP and Ce6-AuNP-Lf. Magnified image: yellow square box in original image. Scale bar: 50 nm. **d** Proportion of hydrodynamic size in Ce6-AuNP and Ce6-AuNP-Lf. **e** Temperature changes of Ce6-AuNP and Ce6-AuNP-Lf under the exposure with 4 W/cm^2^ intensity of PTT (532 nm) laser

PDT and PTT combination therapeutic AuNPs were developed by disulfide conjugation between thiourea-modified Ce6 and the thiol-abundant glutathione coated-AuNP surface. PEGylated-Lf with a thiol-functionalized PEG (Lf-PEG) was incorporated into the AuNP surface by disulfide bonds (Ce6-AuNP-Lf) (Fig. 1d). The FT-IR spectra verified thiourea binding to Ce6 through amide formation (Additional file 1: Figure S1). The first solution was Ce6 modified with thiourea (Ce6-thiol) and the second solution was unmodified Ce6 simply mixed with thiourea (Ce6 + thiourea). The most significant changes in the spectra in the range between 1550 and 1750 cm^−1^ was a band at 1708 cm^−1^, which corresponds to the C=O stretching of carboxylic acids in Ce6 [28]. Ce6-thiol showed a band at 1642 cm^−1^ in the vibrational mode of the amide 1 bond. The new radical formed at 1642 cm^−1^ and the decrease intensity at 1708 cm^−1^ indicate modification of carboxylic acids radicals during amide formation [29]. Next, we tried to prove that Ce6-thiol and PEGylated-Lf were bound to the AuNP surface via Au–S bond through FT-IR spectra. However, it was difficult to identify the newly formed disulfide through adsorption on the AuNP surface because these bands should disappear due to diffusion of the molecules. Instead, to confirm the bond, we detected the specific peak of Ce6 and AuNP. The UV–vis spectrum of the synthesized Ce6-AuNP exhibited specific peaks of Ce6 and AuNP at 400-nm/532-nm and at 671-nm wavelengths, respectively (Fig. 2a). This result demonstrate that it was chemically bound between Ce6-thiol and AuNP by the disulfide bond as intended. Moreover, the binding ratio between Ce6 and AuNP in the Ce6-AuNP was calculated using the absorbance measured at each wavelength and the molar extinction coefficients of AuNP and Ce6. The results showed that an average of 4.5 Ce6 were bound to one AuNP. In addition, our previous study showed that approximately 19.7 GSH-AuNP were bound to one Lf-PEG to make Lf-PEG-AuNP conjugates, which was confirmed by using BCA assay, SDA-PAGE (sodium dodecyl sulfate polyacrylamide gel electrophoresis), and UV–Vis spectra [27].

The surface charge of Ce6, Ce6-AuNP and Ce6-AuNP-Lf were − 9.4 ± 4.3, − 32.7 ± 10.3 and, − 26.8 ± 5.2, respectively (Fig. 2b). From the TEM images, each particle size of Ce6-AuNP and Ce6-AuNP-Lf measured by TEM was about 5 nm (Fig. 2c, see yellow square box). Interestingly, Ce6-AuNP-Lf was well dispersed, while Ce6-AuNP was highly aggregated as shown Fig. 2c. In fact, the aggregated diameters of Ce6-AuNP were 945.2 ± 142.4, 182.1 ± 40.8 and 9.49 ± 12.8 nm, which were within the proportions of 46.4%, 40.8% and 12.8%, respectively (Fig. 2d). The cations present in the solution bound to the carboxylic acid of glutathione coated on the AuNP surface and neutralized the surface charge. This induced irreversible aggregation of AuNP into large structures [30]. However, the aggregated diameters of Ce6-AuNP-Lf were within 7.8 ± 2.6, 15.9 ± 6.2 and 81.1 ± 15.4 nm in proportions of 64.8%, 20.2% and 15.1%, respectively (Fig. 2d). The steric stabilization mediated by PEGylation of Ce6-AuNP-Lf prevented particle aggregation. Maintaining Ce6-AuNP-Lf in sizes from 5 to 20 nm is key for efficient PTT because the surface plasmon resonance (SPR) of AuNP irradiated with PTT laser (532 nm wavelength) is highly reliant on NP size [31]. To evaluate the PTT efficiency of Ce6-AuNP-Lf, 4 W/cm^2^ intensity of PTT laser (532 nm wavelength) was irradiated to them. From the previous literature, it was reported that cancer cell destruction linearly increased in the near-infrared laser intensity range of 1.5–4.7 W/cm^2^ [32]. The maximum temperature (T_max_) of Ce6-AuNP-Lf irradiated with 4 W/cm^2^ intensity of PTT laser for 240 min was 64.5 ± 4.5 °C (Fig. 2e and Additional file 2: Movie S1), while T_max_ of Ce6-AuNP was 43.0 ± 3.7 °C (Fig. 2e and Additional file 3: Movie S2). Likewise, AuNP, which is vulnerable to aggregation between particles, also showed poor heat generation as in Ce6-AuNP. From these results, the photothermal stability of each nanoparticle was calculated as summarized in Table 1. Ce6-AuNP-Lf showed the highest PTT efficiency despite surface modification of AuNP with Lf protein and Ce6 photosensitizer. Also, PEGylated Lf-conjugated AuNP (AuNP-Lf) also showed higher PTT efficiency, suggesting that Lf protein conjugation did not affect the PTT efficiency of AuNP itself. Collectively, we found that steric stabilization by PEGylation of AuNP has a profound effect on PTT efficiency.

**Table 1:** The photothermal stability of AuNP, AuNP-Lf, Ce6-AuNP, and Ce6-AuNP-Lf

	Laser (wavelength, power)	Irradiation time (sec)	Maximum temperature (*T*_*max*_)	PTT efficacy (*T*_*max*_*–T*_*0*_)
AuNP	532 nm, 4 W/cm^2^	240 s	45.4 ± 4.2 °C	20.3 ± 2.1 °C
AuNP-Lf	532 nm, 4 W/cm^2^	240 s	56.3 ± 0.5 °C	29.6 ± 0.2 °C
Ce6-AuNP	532 nm, 4 W/cm^2^	240 s	43.0 ± 3.7 °C	18.4 ± 1.7 °C
Ce6-AuNP-Lf	532 nm, 4 W/cm^2^	240 s	64.5 ± 4.5 °C	39.3 ± 1.3 °C

The temperature changes caused by laser irradiation of AuNP, AuNP-Lf, Ce6-AuNP and Ce6-AuNP-Lf PTT, respectively, measured with a thermal imaging camera

*T*_*max*_, Maximum temperature; *T*_*0*_, Initial temperature

The hydrophobicity of Ce6, Ce6-AuNP and Ce6-AuNP-Lf was assessed through phase transfer between octanol and DW. Here, Ce6-AuNP and Ce6-AuNP-Lf exhibited hydrophilicity despite conjugation with hydrophobic Ce6 (Additional file 1: Figure S2). The AuNP conjugation conferred hydrophilicity that could potentially facilitate muco-penetration in mucus barriers, where the mucin protein constructs hydrophobic bonds with NPs during oral absorption [33]. Furthermore, the surface hydrophobicity of NPs promotes immune recognition by macrophages, allowing the hydrophilic Ce6-AuNP-Lf to reduce undesirable immunostimulation [34]. Therefore, Ce6-AuNP-Lf is expected to be a promising Ce6 delivery carrier to enhance the accumulation of hydrophobic Ce6 in GBM through oral absorption.

### Metal-enhanced reactive oxygen generation (MERos) by conjugation of Ce6 to the AuNP surface

**Fig. 3:**
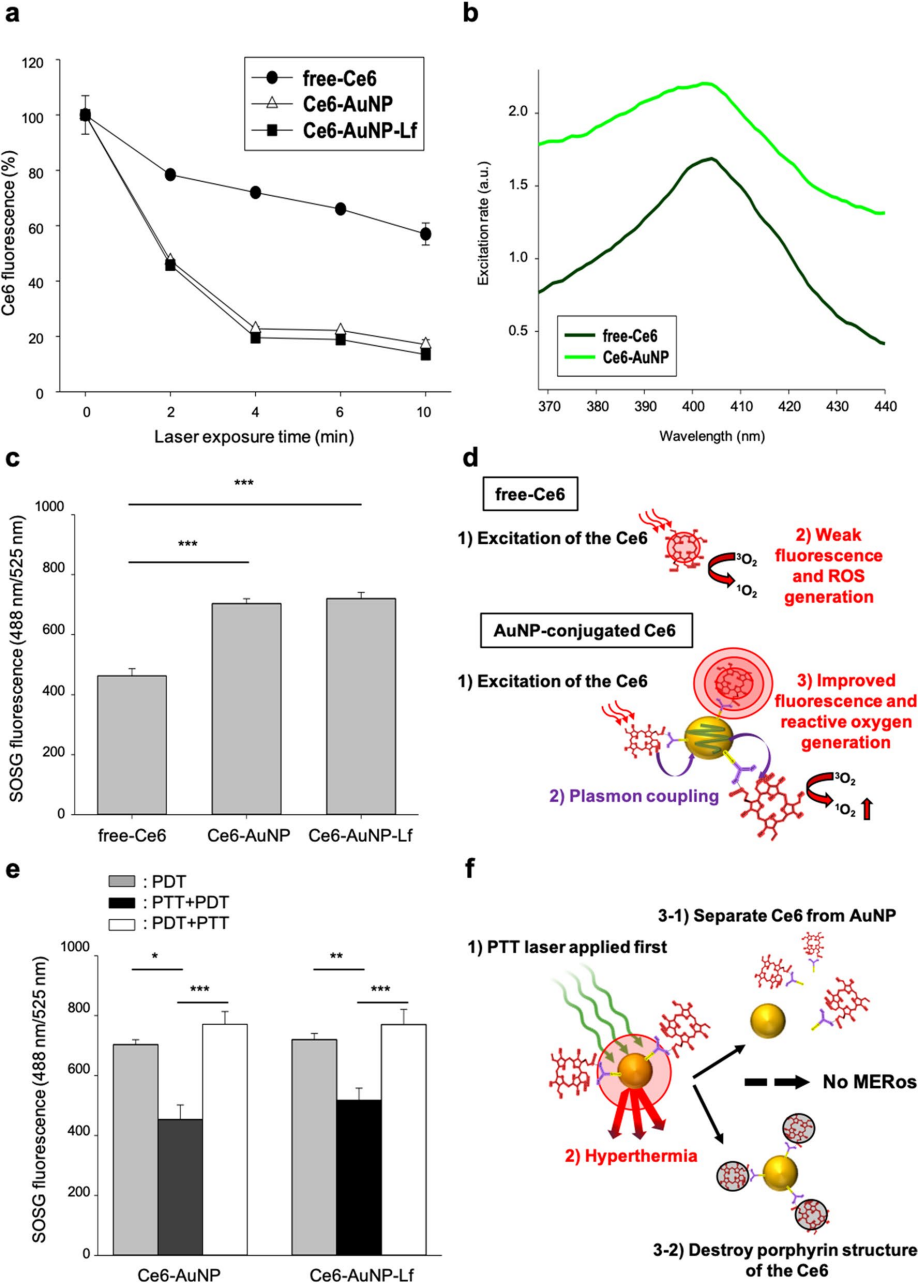
Enhancement of ROS generation ability of AuNP-conjugated Ce6, mediated by MERos.** a** Photobleaching of Ce6 by PDT irradiation in free-Ce6, Ce6-AuNP, and Ce6-AuNP-Lf groups. Data are expressed as mean ± SEM (n = 3). **b** Fluorescence excitation spectra of free-Ce6 and Ce6-AuNP with 2.5 μM Ce6 equivalent concentration. **c** SOSG assay to detect ROS generation by 5 min PDT laser irradiation. Data are expressed as mean ± SEM (n = 3). ***P < 0.001. **d** Schematic illustration of improved fluorescence and ROS generation of Ce6 by plasmon coupling between AuNP and Ce6. **e** Alteration of ability to generate ROS according to laser sequence. The single PDT group was irradiated for 5 min and combined laser groups (PTT + PDT laser order or PDT + PTT laser order) were irradiated for 2.5 min each. Data are expressed as mean ± SEM (n = 3). *P < 0.05, **P < 0.01, ***P < 0.001. **f** Schematic illustration of the lost MERos effect by first applying the PTT laser in the PTT + PDT laser order

During 10 min of PDT laser irradiation, the Ce6 fluorescence of free-Ce6, Ce6-AuNP and Ce6-AuNP-Lf gradually decreased to 57.0 ± 5.6%, 17.1 ± 2.5% and 13 ± 2.6%, respectively (Fig. 3a). Due to photobleaching, fluorescence decreased with light irradiation by a fourfold larger change in Ce6-AuNP and Ce6-AuNP-Lf compared to free-Ce6. This is due to MEF by plasmon coupling between AuNP and Ce6, which improves fluorescence intensity but triggers photobleaching [35]. This process is explained by the MEF rather than the FRET of the distance-dependent energy transfer process between the two fluorophores. Since AuNPs do not act as fluorophores, unlike Ce6, metal nanoparticles such as Au, Ag, Cu, and Pt are more suitable as MEFs that increase the fluorescence intensity of fluorophores [36]. In addition, from the UV–vis spectrogram, AuNP-conjugated Ce6 (Ce6-AuNP) showed a 1.3-fold improved excitation compared to free Ce6 in the fluorescence excitation spectrum (Fig. 3b). This demonstrated the role of AuNP as a nanoantenna that increased the excitation energy of Ce6, which can either induce fluorescence or generate ROS [37]. In fact, the decrease of Ce6 fluorescence via photoexcitation is related to ROS generation [38]. This is because the generated ROS depletes the remaining Ce6, reducing fluorescence [39]. Consistent with the correlation between ROS generation and fluorescence reduction, in our results, PDT irradiation generated 1.6-fold more ROS in the AuNP-conjugated Ce6 (Ce6-AuNP and Ce6-AuNP-Lf) than with free-Ce6 (Fig. 3c). Therefore, MEF and ROS production in AuNP-conjugated Ce6 such as Ce6-AuNP and Ce6-AuNP-Lf is characterized by simultaneous increase (Fig. 3d). There are several papers demonstrating the correlation between MEF and ROS production between AuNP and PSs. [24, 40] The mechanisms of the relationship between MEF and MERos are expected to be due to inter-system crossover between AuNP and PSs, which promotes the triplet state of PSs [41]. In this regard, the proximity of Ce6 to the gold ions on the AuNP surfaces enhanced spin–orbit coupling due to the external heavy atom effect [42, 43], increasing triplet formation.

Moreover, Ce6-AuNP and Ce6-AuNP-Lf had significant differences in ROS generation ability according to the application sequence of PDT and PTT (Fig. 3e). As a result, ROS generation was lowest in the PTT + PDT (applying PTT then PDT). However, it was rather effective in single PDT and PDT + PTT (applying PDT then PTT). This can be explained as follows. In the case of PTT + PDT (applying PTT then PDT), (1) AuNPs generate high heat in response to the first applied PTT laser. (2) Then, Ce6 probably separates from the AuNP surface or destroys the porphyrin structure of Ce6. (3) MERos no longer exist due to loss of plasmon coupling between Ce6 and AuNP. (4) Therefore, ROS generation of the PTT + PDT laser group was relatively lower than that of single PDT or the PDT + PTT laser group (Fig. 3f).

### Stability of Ce6-AuNP in an oral absorption- mimicking environment

To evaluate the colloidal stability of Ce6-AuNP-Lf during the oral absorption procedure, we first mimicked the pH environments of the gastric and intestinal system. At pH 2, the absorbance of Ce6-AuNP-Lf was slightly decreased at 532 and 671 nm, but there was no peak shift (Additional file 1: Figure S3a). At pH 5, no peak shift or decrease in absorbance were observed until 24 h (Additional file 1: Figure S3b). To more specifically simulate the oral absorption procedure of Ce6-AuNP-Lf, it was serially exposed to pH 2 for 3 h and then pH 5 for an additional 12 h (Additional file 1: Figure S3c) [44]. Ce6-AuNP-Lf showed stability without peak shift and decreased absorbance in both the 532 and 671 nm wavelength bands. As a result, Ce6-AuNP-Lf maintained the colloidal stability of AuNP and undesired release of Ce6 was not detected at the pH conditions of the GI tract. Furthermore, Ce6-AuNP-Lf was stable in both physiological solution of PBS and of 10% FBS for 180 days (Additional file 1: Figure S4a and 4b). Based on these findings, we conclude the suitability of Ce6-AuNP-Lf as an oral formulation.

### Enhanced permeability of Ce6-AuNP-Lf at the epithelial barriers of the GI tract

**Fig. 4:**
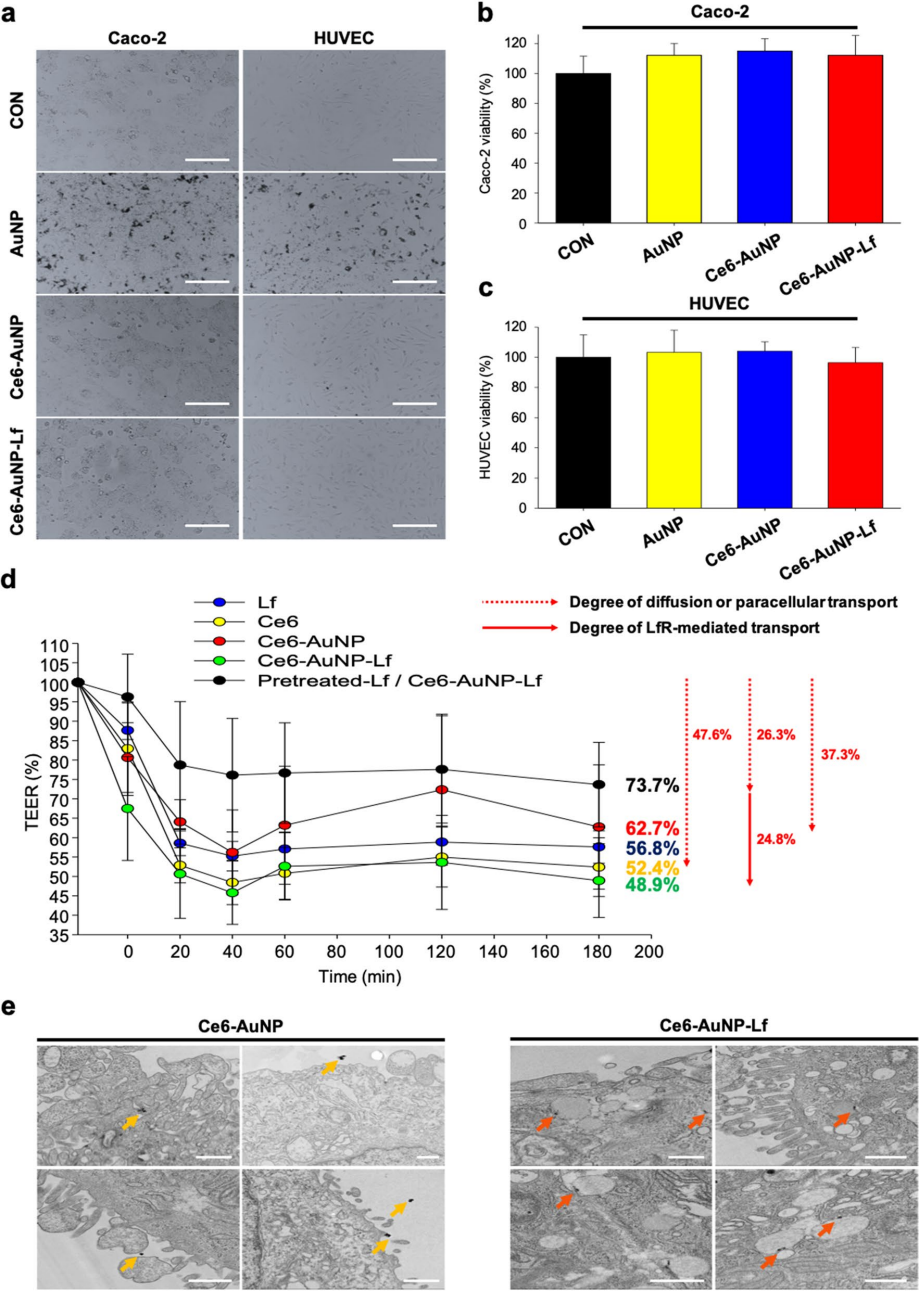
In vitro small intestine epithelial (Caco-2) cytotoxicity and permeability study of Ce6-AuNP-Lf.** a** Morphology of small intestinal Caco-2 cells and human umbilical vein endothelial cells (HUVECs) after treatment with AuNP, Ce6-AuNP and Ce6-AuNP-Lf for 24 h. Magnification: × 100. Scale bar: 100 μm. **b** The viability of Caco-2 cells after treatment with AuNP, Ce6-AuNP, and Ce6-AuNP-Lf, respectively, for 24 h. Data are expressed as mean ± SEM (n = 8). **c** The viability of HUVECs after treatment of AuNP, Ce6-AuNP and Ce6-AuNP-Lf, respectively, for 24 h. Data are expressed as mean ± SEM (n = 8). **d** TEER value through Caco-2 cell monolayer after treatment of each group. Data are expressed as means ± SEM (n = 4). **e** TEM images of Caco-2 cell monolayers treated with Ce6-AuNP and Ce6-AuNP-Lf. Yellow arrows: Ce6-AuNP. Orange arrows: Ce6-AuNP-Lf internalized through LfR endocytosis. Scale bar: 500 nm

In oral absorption of drugs, the administered AuNP, Ce6-AuNP and Ce6-AuNP-Lf penetrate through the small intestine into the blood. Therefore, the toxicities of AuNP, Ce6-AuNP and Ce6-AuNP-Lf were examined using Caco-2 cells and human umbilical vein endothelial cells (HUVECs). Treatment with NPs did not cause significant cytotoxicity and morphological changes in Caco-2 cells and HUVECs (Fig. 4a–c). Intestinal epithelial cells, including Caco-2 cells, express LfR on their membranes [45–47]. Thus, the Caco-2 cell permeability test was conducted to determine whether the permeation of Ce6-AuNP-Lf was increased by LfR in the GI tract. Furthermore, the TEER values decreased with drug transmittance in the Caco-2 cell monolayer. As a result, the TEER values of the Ce6, Ce6-AuNP and Ce6-AuNP-Lf groups decreased to 52.4%, 62.7%, and 48.9% respectively (Fig. 4d).

In general, two routes (transcellular and paracellular pathways) are available for transport through the Caco-2 cell barrier. The transcellular route of barrier is employed by lipophilic drugs and by molecules selectively transported by receptors, channels, pumps and carriers present in the plasma membrane. Therefore, transmittance of hydrophobic Ce6 (~ 47.6%) can be interpreted as the result of transcellular diffusion into the cellular plasma membrane. However, the hydrophilic Ce6-AuNP and Ce6-AuNP-Lf also were transported through the barrier in amounts of 37.3% and 51.1% respectively. In fact, AuNP treatment is known to increase the paracellular pathway in the in vitro epithelial and endothelial permeability assay. This is known to be mediated by suppression of threonine phosphorylation on occludin and zonula occludens-1 (ZO-1), and of perturbed occludin/ZO-1 complex formation and disassembly of tight junctions [48, 49]. Therefore, Ce6-AuNP and Ce6-AuNP-Lf were able to cross the Caco-2 cell barrier through the paracellular pathway at the tight junctions. Furthermore, we speculated that the enhanced permeability of Ce6-AuNP-Lf (~ 13% permeability) compared to Ce6-AuNP was due to the LfR-mediated transcytosis in the Caco-2 cell barrier. In general, pinocytosis is known to uptake small AuNPs (< 500 nm) by forming protrusions on the cell surface, but the corresponding process was not verified in the Bio TEM results after treatment with Ce6-AuNP [50]. In contrast, intracellular entrapment of Ce6-AuNP-Lf in vesicles was observed, which explains LfR-mediated transcytosis in Caco-2 cells (Fig. 4e).

The pretreated-Lf/Ce6-AuNP-Lf group was used to verify only the passive transport of Ce6-AuNP-Lf by excluding the LfR-mediated transport. The LfRs on the Caco-2 cell monolayer were fully occupied with free Lf in advance and then Ce6-AuNP-Lf was further treated (Additional file 1: Figure S5). The difference in transmittance between the Pretreated-Lf / Ce6-AuNP-Lf group and Ce6-AuNP-Lf group was 24.8%. This demonstrates that 24.8% of Ce6-AuNP-Lf are transported through LfR-mediated transcytosis and the remaining 26.3% are passively transported through a LfR-independent route, as with Ce6-AuNPs (Fig. 4d).

### Ce6-AuNP-Lf permeates the BBB by LfR-mediated transcytosis and accumulate in GBM cells, resulting in severe phototoxicity by the MERos effect

**Fig. 5:**
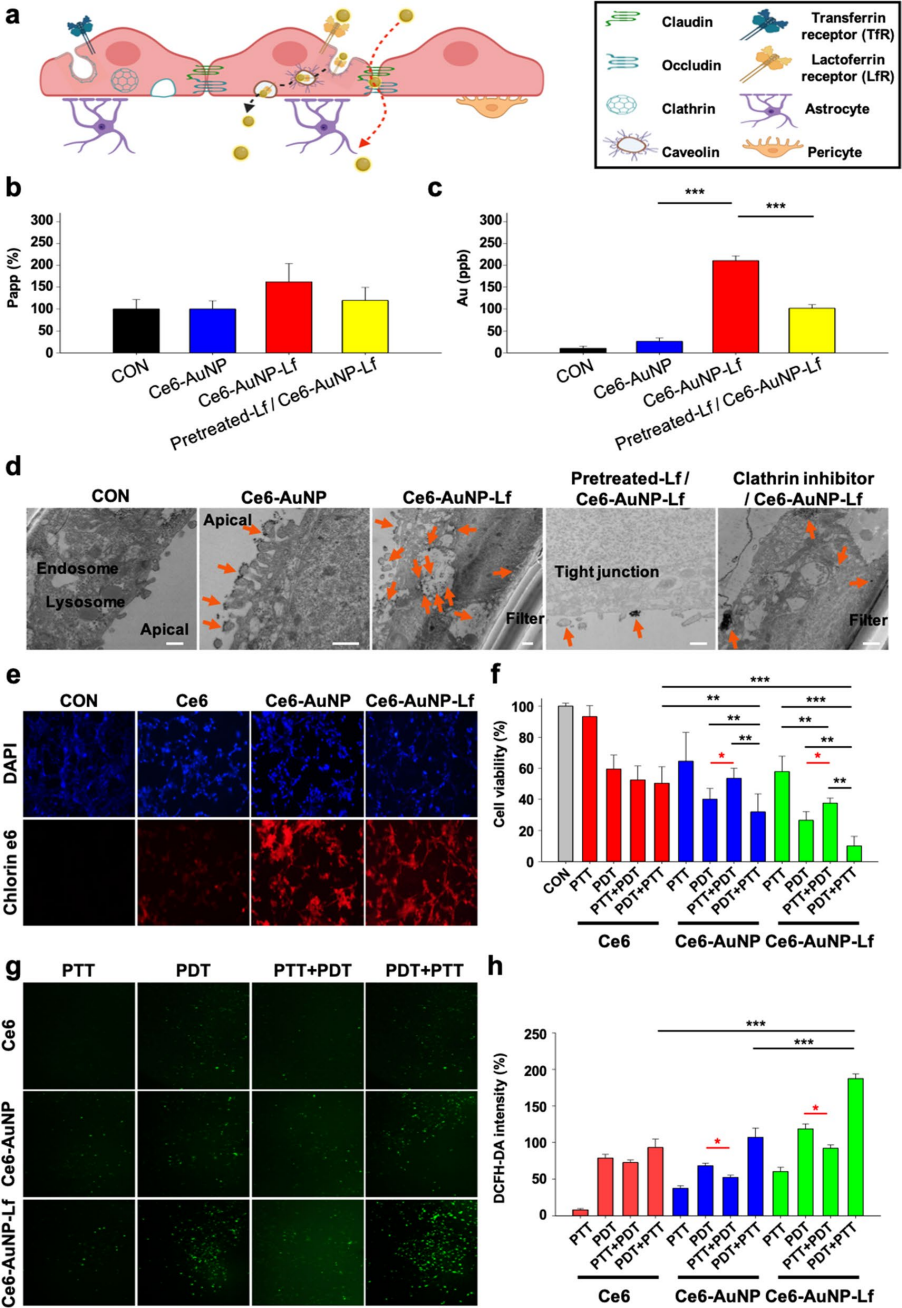
In vitro Ce6-AuNP-Lf efficiently crosses the BBB barrier and sequentially accumulates in GBM cells to drive therapeutic outcomes. **a** Schematic illustration of the BBB model for permeability test. The illustration was created with BioRender.com. **b** Apparent permeability (P_app_) using FITC-Dextran to evaluate integrity of tight junctions. Data are expressed as the mean ± SEM (n = 3). **c** The absolute amount of gold nanoparticles passing through the semi-permeable BBB model as measured by ICP-MS. Data are expressed as the mean ± S.E.M (n = 3). ***P < 0.001. **d** TEM images of the drug-treated BBB model; Orange arrows indicate AuNP **e** Confocal images of U87MG after 24 h treatment with Ce6, Ce6-AuNP and Ce6-AuNP-Lf, respectively. **f** Reduced U87MG cell viability by phototoxicity. Data are expressed as the mean ± SEM (n = 4). *P < 0.05, **P < 0.01, ***P < 0.001. **g** Intracellular DCFH-DA fluorescence signals (excitation / emission at 485 nm / 535 nm) after each treatment. (h) U87MG intracellular ROS generation by phototoxicity. Data are expressed as the mean ± SEM (n = 4). *P < 0.05, **P < 0.01, ***P < 0.001

The permeability of Ce6-AuNP-Lf in BBB was examined using a human BBB Transwell model [51]. The BBB model was prepared by culturing human brain microvascular endothelial cells on the apical side of the insert interfaced with primary human pericytes and astrocytes **(**Fig. 5a). TEER value, an indicator of development of tight junction, was measured to determine the barrier function of BBB. The BBB endothelium showed a physiological level of TEER value (average 4079 Ω·cm^2^), indicating that the BBB model can provide very limited paracellular transport. The expression of transport systems including functional efflux pump and receptors such as LfR and transferrin receptor (TfR) was previously demonstrated [51].

We measured the amount of AuNPs that crossed the BBB using the human BBB Transwell model. To monitor the amount of AuNPs across the BBB during the assay, apparent permeability (P_app_) of FITC-Dextran (3 kDa) was measured to evaluate the integrity of tight junctions (Fig. 5b). The concentration of Ce6-AuNP-Lf in the basal chamber of Transwell was 8.13 times higher than the concentration of Ce6-AuNP indicating that functionalization with Lf significantly enhanced BBB penetration efficacy without breakdown of barrier function (Fig. 5c). Bio TEM images showed that Ce6-AuNP-Lf was internalized by brain endothelial cells packed by vesicles implying the LfR-mediated endocytosis of Ce6-AuNP-Lf (Fig. 5d). A competitive assay was conducted to assess qualitative binding information of Ce6-AuNP-Lf to the LfR expressed on BBB which can lead to transcytosis of NPs. Pretreatment with Lf to a BBB model decreased BBB penetration of Ce6-AuNP-Lf by 52%, which demonstrates that BBB penetration of Ce6-AuNP-Lf is dependent on the interaction between Lf and LfR (Fig. 5c and Additional file 1: Figure S5). The Bio-TEM images of Ce6-AuNP-Lf also revealed that Ce6-AuNP-Lf is not internalized by brain endothelial cells in the presence of Lf. To examine the endocytic mechanism of Ce6-AuNP-Lf, it was monitored using Bio TEM in the presence of the inhibitor of clathrin-medicated endocytosis. The Ce6-AuNP-Lf was still detected inside the vesicles even with the blocking of clathrin-mediated endocytosis, which indicates that Ce6-AuNP-Lf can be internalized by cells through clathrin-independent endocytosis (Fig. 5d). The mechanism of transcytosis of Lf through BBB remains controversial. There is evidence that internalization of NPs functionalization with Lf is partially (40%) decreased by inhibitors of clathrin-mediated endocytosis mannitol (200 mM) [52], which is not consistent with our results. However, the endocytic pathway also depends on the geometrical, physical, and chemical properties of NPs. It is assumed that physical and chemical properties of Ce6-AuNP-Lf resulted in high dependence of a non-clathrin-mediated uptake pathway.

Confocal microscope images of GBM cells show that AuNP improved insufficient tumor accumulation of Ce6. The intracellular fluorescence of Ce6 was significantly increased in the Ce6-AuNP and Ce6-AuNP-Lf groups compared to that of free-Ce6 (Fig. 5e). The amounts of Ce6, Ce6-AuNP and Ce6-AuNP-Lf uptake into the cells were 43.6 ± 2.2, 61.3 ± 1.6 and 82.1 ± 2.1, respectively (Additional file 1: Figure S6). The cell uptake of Ce6-AuNP-Lf was statistically increased compared to that of Ce6-AuNP, which lacks Lf as a targeting ligand. This indicates enhanced GBM cell uptake for targeted conjugates compared to un-targeted conjugates [53]. Due to the high accumulation of Ce6-AuNP-Lf, they further reduced cell viability by phototherapy compared to the Ce6 and Ce6-AuNP groups. The apoptotic cell population (Annexin-V + /PI +) of Ce6-AuNP-Lf, Ce6-AuNP and Ce6 groups to which the PDT + PTT was applied were 45.9%, 26.5%, and 22.0% of the whole, respectively (Additional file 1: Figure S7). Furthermore, PDT + PTT (applying PDT then PTT) showed a superior therapeutic effect compared to other treatments such as PTT + PDT (applying PTT then PDT) and single PDT or PTT. Despite the two types of treatment applications, PTT + PDT (applying PTT then PDT) in the cells treated with Ce6-AuNP and Ce6-AuNP-Lf was less effective than single PDT (Fig. 5f). This is because MERos was absent in Ce6 conjugated to AuNP by the first-applied PTT (Fig. 3e and 3f). Likewise, intracellular ROS fluorescence by the cells treated with Ce6-AuNP or Ce6-AuNP-Lf was lower in PTT + PDT (applying PTT then PDT) than in the single PDT but highest in PDT + PTT (applying PDT then PTT) (Fig. 5g and h). Briefly, when PTT was applied first, Ce6-AuNP and Ce6-AuNP-Lf, in which Ce6 is bound to AuNP, lost the MERos effect, resulting in decreased ROS production. Therefore, the therapeutic effect of PTT + PDT (applying PTT then PDT) was lower than that of PDT + PTT (applying PDT then PTT) and PDT alone.

### Improved oral bioavailability and GBM targeting of Ce6-AuNP-Lf in orthotopic GBM mice

**Fig. 6:**
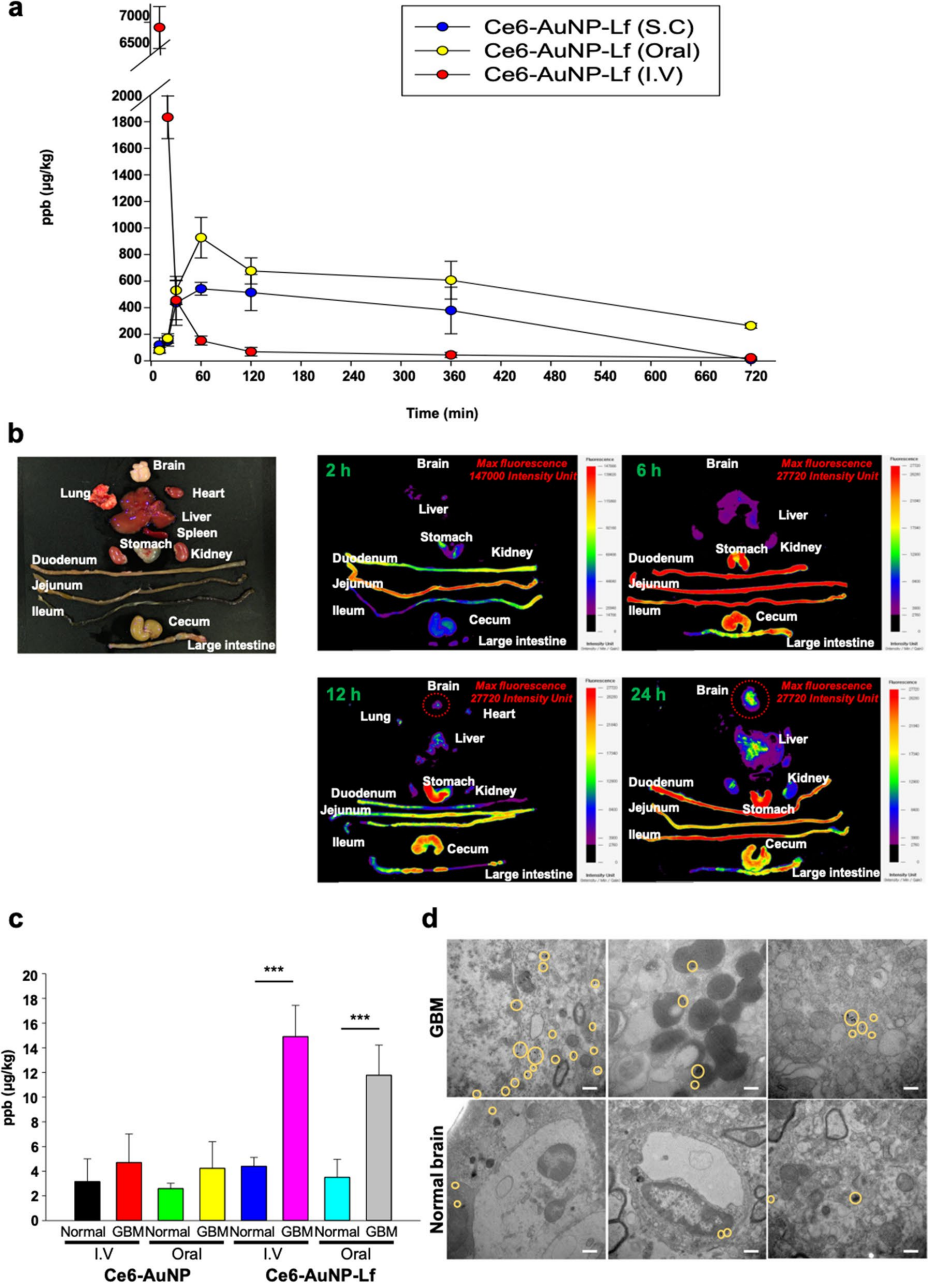
In vivo oral absorbable and GBM targeting evaluation of Ce6-AuNP-Lf. **a** Pharmacokinetics of Ce6-AuNP-Lf administered at 30 mg/kg, 5 mg/kg, and 60 mg/kg subcutaneously (SC), intravenously (IV) and orally, respectively. Data are expressed as mean ± SEM (n = 3) **b** Fluorescence tracer image of orally administered Ce6-AuNP-Lf. Intensity unit indicates the intensity / min / gain. Red dashed line indicates the detected fluorescence signal in the brain. **c** AuNP accumulation in the normal brain and GBM after 24 h administration of Ce6-AuNP and Ce6-AuNP-Lf via IV and oral, respectively. Data are expressed as mean ± SEM (n = 4). ***P < 0.001. **d** Bio-TEM images of GBM and normal brain after 24 h oral administration of 60 mg/kg Ce6-AuNP-Lf. Yellow circles indicate the accumulated AuNP. Scale bar: 500 nm

The influx and clearance of Ce6-AuNP-Lf in blood were investigated after oral, subcutaneous, and intravenous administration, respectively (Fig. 6a). As a result, the half-lives (T_1/2_) of oral and SC administered groups were 521 ± 20 min and 395 ± 10 min, respectively (Table 2). These remarkable blood residence results of Ce6-AuNP-Lf were due to the short PEG (232 Da) coating on the AuNP surface. In general, incorporation of PEG on the AuNPs enhanced the absorption of nanoconjugates to systemic circulation and increased its residence time in blood circulation [54]. Shorter PEG chains increased the absorption efficiency when administered orally compared to the longer chain [55]. Furthermore, SC injection a sustained diffusible method among the various drug injection route. This is due to the large amount of capillaries in the fatty layer of subcutaneous tissue just beneath the skin [56]. However, the mean retention time (MRT) of orally administered Ce6-AuNP-Lf was better than that of SC. In addition, the percentages of bioavailability (F_abs_) were similar at 8.6 ± 1.2% and 7.3 ± 0.6% in the SC group and the oral group, respectively. The fluorescence signal was maximum in the fluorescence tracer image of orally administered Ce6-AuNP-Lf at 2 h with 147,000-intensity units (I.U.) in the jejunum, where LfR was overexpressed (Fig. 6b) [57]. The IU of the fluorescence then decreased to 27,720 after 6 h, indicating that the orally administered Ce6-AuNP-Lf first passes from the intestine to the liver before reaching systemic blood circulation. Therefore, the fluorescence signal in the liver continued to increase up to 24 h. Notably, the brain fluorescence signal was found after 12 h and increased to about 13,000 I.U. at 24 h after oral administration. It can be inferred that orally administered Ce6-AuNP-Lf is stably absorbed with high efficiency in the GI tract. Then, it subsequently flows into the blood through the capillaries of the small intestine and is able to overcome the BBB barrier to reach the brain tissue. The intestinal barrier permeability test also demonstrated that Ce6-AuNP-Lf improved overall transmittance through LfR-mediated transport as well as passive transport (Fig. 4d). Furthermore, the colloidal stability of Ce6-AuNP-Lf was maintained under severe pH conditions of the GI tract (Additional file 1: Figure S3) and in the presence of serum (Additional file 1: Figure S4). Therefore, outstanding pharmacokinetics characteristics of the orally administered Ce6-AuNP-Lf were obtained.

**Table 2:** Pharmacokinetics parameters of Ce6-AuNP-Lf administered subcutaneously (SC), intravenously (IV), and orally

Route	Dose (mg/kg)	T_max_ (min)	C_max_ (μg/mL)	C_0_	V_d_	T_1/2_ (min)	AUC_0-720_ (μg min/kg)	F_abs_ (%)
Subcutaneous (S.C.)	30	60	0.5 ± 0.1	–	–	395 ± 10	227,608 ± 3,254	8.6 ± 0.6
Oral	60	60	0.9 ± 0.2	–	–	521 ± 20	385,450 ± 2,346	7.3 ± 1.2
Intravenous (I.V)	5	0	62.5	62.5	80	5.6 ± 3.1	443,054 ± 4,410	100

Periodic gold concentration in blood after administration of Ce6-AuNP-Lf subcutaneously, orally and intravenously, respectively, were measured by ICP-MS. Data are expressed as means ± SEM (n = 3). AUC_0-720_, area under the plasma concentration curve from hour 0 to 720; C_max_, maximum drug concentration in plasma; C_0_, initial drug concentration; V_d_, volume of distribution; F_abs_, absolute bioavailability; t_1/2_, half-life; t_max_, time to C_max_

Next, we compared the GBM targeting efficacy of Ce6-AuNP and Ce6-AuNP-Lf that were administered by IV and oral, respectively. IV administration of Ce6-AuNP-Lf showed accumulation at 14.9 ± 2.2 μg/kg and 4.3 ± 0.6 μg/kg in GBM and normal brains, respectively. By oral administration, it accumulated at 11.8 ± 2.1 μg/kg in GBM and 3.5 ± 1.3 μg/kg in the normal region (Fig. 6c). In the bio-TEM result of brain tissue collected 24 h after oral administration, more Ce6-AuNP-Lf was found at the GBM compared to in normal brain regions (Fig. 6d). Unexpectedly, difference in AuNP accumulation between GBM and normal brain regions were found in Ce6-AuNP administration. For instance, in IV administration of Ce6-AuNP, it accumulated at 4.7 ± 2.0 μg/kg and 3.2 ± 1.6 μg/kg in GBM and the normal brain, respectively. In oral administration, it accumulated at 4.2 ± 1.9 μg/kg and 2.6 ± 0.4 μg/kg in GBM and normal brain, respectively (Fig. 6c). This can be interpreted as the result of the EPR effect, which is commonly caused by abnormal angiogenesis and tumor pressure in GBM [58]. However, the amounts of NPs that penetrated into the BBB and accumulated in the GBM were significantly higher for Ce6-AuNP-Lf, which demonstrates that GBM targeting is dependent on the interaction between Lf and LfR expressed in BBB and GBM.

To further confirm the GBM targeting of Ce6-AuNP-Lf and the presence of non-specific drug distribution, a PTT laser was applied at the GBM site and the body flank after 24 h oral administration. The excellent targeting effect of the Ce6-AuNP-Lf on GBM was identified through a temperature increase to 54.2 ± 0.1 °C. At the same time, the temperature rise on the body flank was 37.6 ± 0.4 °C due to reduction of the non-specific distribution by high targeting to GBM (Additional file 1: Figure S8). Therefore, the remarkable GBM targeting of Ce6-AuNP-Lf proved to be suitable as a GBM therapy.

To achieve successful phototherapy in the GBM of the brain using Ce6-AuNP-Lf, the PTT laser must pass through several barriers such as the scalp, skull, and brain tissue. In general, treatment using near-infrared rays is known to be effective up to ~ 3 mm below the skin. However, in this study, when near-infrared (NIR) ray was irradiated to the head of an experimental animal, the temperature of the head area increased to an effective level (Additional file 1: Figure S8). These results were thought to be possible because the average thickness of the mouse skull is 0.34 ± 0.05 mm [59]. In addition, in order to clearly evaluate whether the transmitted PDT laser can have an energy level capable of generating ROS from AuNPs and Ce6-AuNP-Lf, a PDT laser (671 nm) was irradiated to the outside of mouse skin tissue (1 cm thickness) (Additional file 1: Figure S9). As a result, the ROS generation of Ce6-AuNP and Ce6-AuNP-Lf was similar to that when irradiated directly without tissue intervention, suggesting that the PDT laser energy penetrated through the thicker skin tissue is sufficient to generate ROS in Ce6-AuNP and Ce6-AuNP-Lf due to the MERos advantage in our system. However, from a clinical point of view, these results in experimental animal models are difficult to apply directly to clinical trial because the thickness of a human scalp is about 6 mm and the thickness of a skull is about 6–7 mm. Therefore, to be effectively applied in clinical trial, Ce6-AuNP-Lf with the advantages of MERos should be equipped with optimized nanoparticles or photosensitizers sensitive to long wavelengths (> 1000 nm) that are more favorable for tissue penetration. In fact, the possibility of brain imaging using the long-wavelength NIR region (1.3–1.4 um) in an experimental animal model was recently reported from other literature [60].

### In vivo* PDT and PTT of Ce6-AuNP-Lf in orthotopic GBM mice model*

**Fig. 7:**
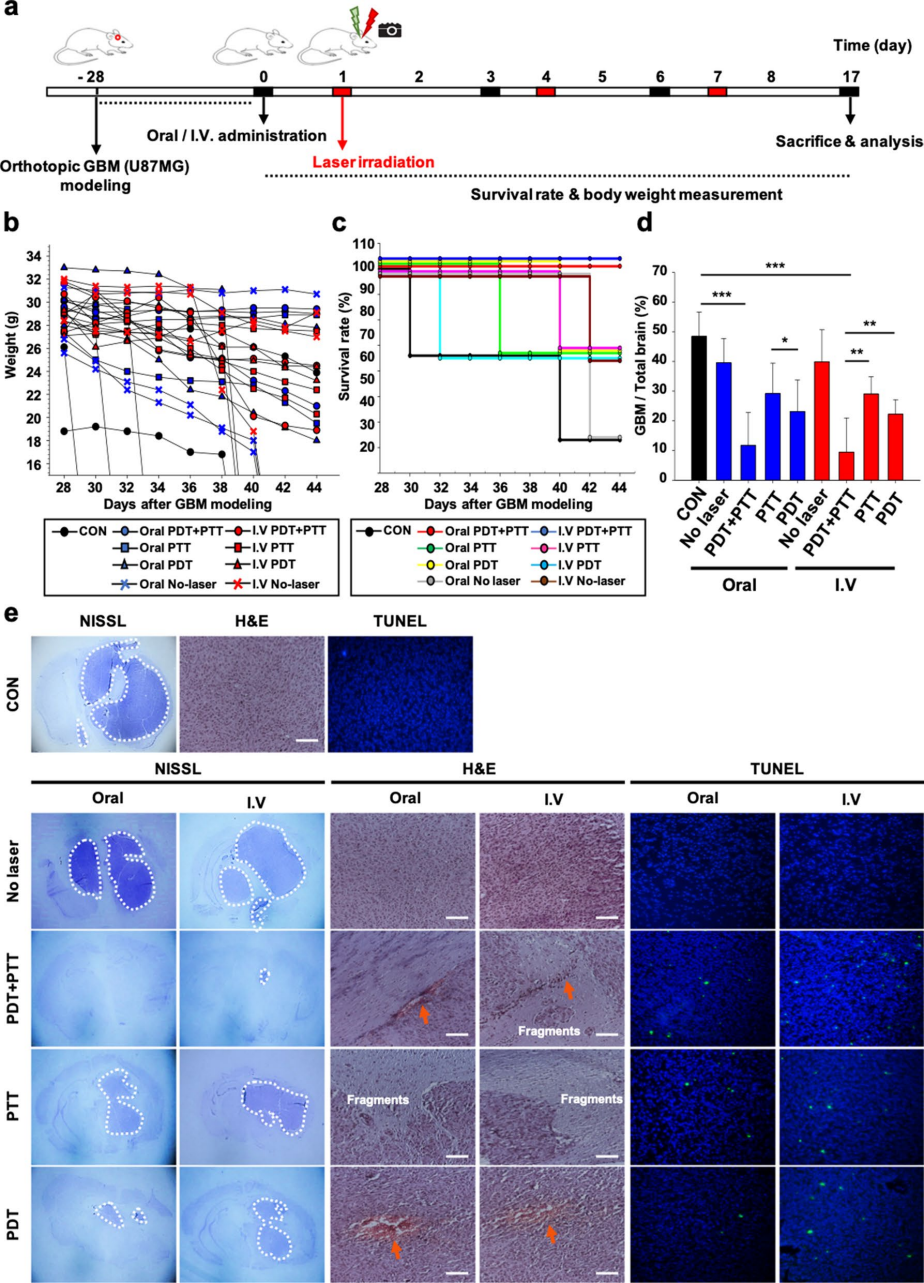
In vivo PDT and PTT therapeutic effects of Ce6-AuNP-Lf in an orthotopic U87MG derived-GBM mice model.** a** Schematic illustration of treatment cycle including drug administration, laser irradiation and monitoring of survival rate and body weight transformation. **b** Weight transformation. Data are expressed as mean ± SEM (n = 5). **c** Survival rate during treatment until day 44. Data are expressed as mean ± SEM (n = 5). **d** Proportion of GBM in the brain. Data were expressed as mean ± S.E.M (n = 5). *P < 0.05, **P < 0.01, ***P < 0.001. **e** Nissl, H&E and TUNEL staining of mice brain. White dashed lines indicate remaining GBM. Orange arrows indicate thrombosis. Scale bar: 50 μm

After in vivo GBM targeting assessment, the therapeutic photothermal and photodynamic effects of Ce6-AuNP-Lf that accumulated in GBM were evaluated. Laser irradiation was applied to the GBM-induced region of mice 24 h after administration. The treatment cycle including Ce6-AuNP-Lf administration and laser irradiation was repeated 3 times. The survival rates and weight changes were measured until sacrifice (Fig. 7a). As a result, the group to which PDT + PTT was applied after oral or IV administration maintained the body weight from the beginning of treatment (Fig. 7b) and showed a 100% survival rate until the last day (Fig. 7c). Weight loss is one of the parameters of repeated-dose toxicology [61]. Therefore, it can be inferred that repeated administration of Ce6-AuNP-Lf itself does not have systemic toxicity. After all treatments were completed, the mice were sacrificed to assess the therapeutic effect through histological studies. The proportion of GBM in the total brain tissue was determined through Nissl staining. As a result, the PDT + PTT group reduced the GBM proportion to 11.8 ± 9.8% and 9.5 ± 10.3% in oral and IV administration, respectively. On the other hand, the No-laser, PTT and PDT groups decreased to 39.5 ± 7.3%, 29.2 ± 9.2% and 23.1 ± 9.5%, respectively, with the oral administration. Furthermore, in IV administration, GBM proportion decreased to 39.9 ± 9.6%, 29.1 ± 5.2% and 22.2 ± 4.7%, respectively (Fig. 7d and e). This suggests that the PDT + PTT combination treatment, the most potent treatment strategy by MERos of Ce6-AuNP, is consistent in the orthotopic GBM model.

To evaluate the therapeutic effect of Ce6-AuNP-Lf with PDT + PTT combination treatment, the extracted brain tissues were immunohistolotically analyzed. The no-laser group and control group exhibited 80–100% intact tumor cells in H&E results (Fig. 7e, H, E). The tumor tissue has a dense structure and consists of polymorphic cells 10 to 20 μm in diameter and oval nuclei. However, in groups irradiated with the laser, the proportion of intact tumor cells decreased, and various types of cell death were observed. In the groups containing PTT laser irradiation, there were fragments separated from solid tumors. In this case, the PTT laser irradiation is believed to cause apoptosis by physically damaging the tumor cells via Ce6-AuNP-Lf hyperthermia. In addition, more TUNEL-positive apoptotic cells were found in the PTT group than in the PDT group (Fig. 7e, TUNEL). Thus, the ROS generated from Ce6-AuNP-Lf by PDT laser irradiation caused cancer cell death. Furthermore, we found that there was no any damage at the normal brain tissue from the results of H&E and TUNEL stain after PDT + PTT combination treatment. These results demonstrated that 4 W/cm^2^ intensity of laser do not affect the normal brain tissue. However, to clearly evaluate the effect of PDT + PTT laser, it is necessary to further optimize the intensity and irradiation time of PDT + PTT laser for precisely modulating the generating ROS and temperature.

### Tumor regression and pro-inflammatory cytokine increase due to phototherapy of Ce6-AuNP-Lf in an orthotopic GBM rat model

**Fig. 8:**
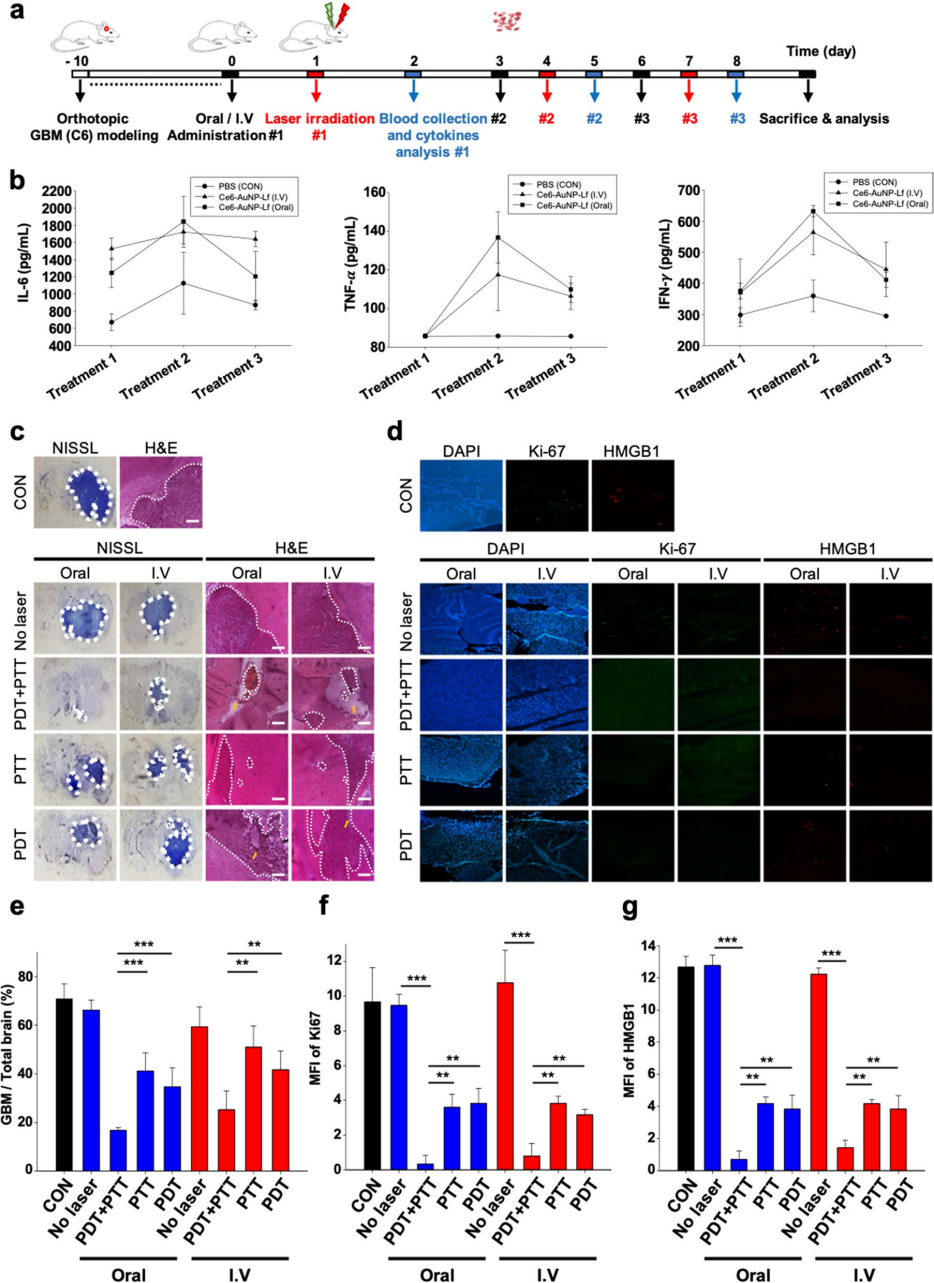
In vivo PDT and PTT therapeutic effects of Ce6-AuNP-Lf and occurrence of pro-inflammatory cytokines in the orthotopic C6 glioma rat.** a** Schematic illustration of treatment cycle including drug administration, laser irradiation and blood collection for cytokines analysis. **b** ELISA of pro-inflammatory cytokines IL-6, TNFα, and IFN-γ from the blood collected after treatment #1, #2 and #3. Data are expressed as mean ± SEM (n = 5). **c** Nissl and H&E staining of the brain. White dashed lines indicate remaining GBM regions. Yellow arrows indicate tumor necrosis. Scale bar: 50 μm. **d** Immunofluorescence staining of Ki67 and HMGB1 in GBM tissues after all treatment cycles. **e** Proportion of GBM in the brain. Data are expressed as mean ± SEM (n = 5). **P < 0.01, ***P < 0.001. **f** Quantification of Ki67 mean fluorescence intensity (MFI). Data are expressed as mean ± SEM (n = 5). **P < 0.01, ***P < 0.001. **g** Quantification of HMGB1 MFI. Data are expressed as mean ± SEM (n = 5). **P < 0.01, ***P < 0.001

We constructed a GBM rat model using the C6 glioma cell to determine whether our treatment strategy is effective in a variety of orthotopic models. Unlike nude mice that do not have an immune system [62], rats have a system that can be activated by PDT + PTT. The influence of PDT and PTT on the immune response involves acute inflammation, leukocyte infiltration of the tumor, and production of pro-inflammatory cytokines [63]. Thus, blood was collected periodically and investigated using the pro-inflammatory cytokine that is released into serum by PDT + PTT treatments (Fig. 8a). After treatment #1, the IL-6 was increased in the peripheral blood in both IV and oral administration groups. However, IFN-γ and TNFα were not elevated. After treatment #2, the levels of all three cytokines (IL-6, IFN-γ, and TNFα) exponentially increased. Those cytokines were present at the highest concentration after treatment #2 and then decreased in treatment #3 but maintained higher levels compared to the CON group (Fig. 8b). The phototherapy (PDT and PTT) induced cell death generated a strong and acute local inflammatory response at the treated sites to attack tumor cells [64, 65]. This immune system involves the expression of NF-κB transcription factors which lead to cytokines release. Therefore, upregulation of pro-inflammatory cytokines (IL-6, IFN-γ, and TNFα**)** during the treatment cycle can be inferred because PDT + PTT of Ce6-AuNP-Lf subsequently destroying GBM due to the immune response.

As a result of the histology study, the combination laser therapy (PDT + PTT) was the most effective in reducing the GBM proportion in the brain. GBM decreased to 16.7 ± 1.1% and 25.2 ± 6.9% in oral and IV administration, respectively. Furthermore, PDT + PTT irradiation induced apoptosis and necrosis in GBM, leading to the greatest amount of tumor destruction (Fig. 8c and e, nissl staining). Consistent with the results of a GBM mice model, hemorrhage and thrombosis were observed in the groups exposed to PDT laser irradiation (Fig. 8c, H, E). ROS generated by PDT causes irreversible damage in endothelial cells and the vascular membrane. Since tumor growth is related to vasculature function due to the oxygen and nutrient supply, microvasculature destruction by PDT damages tumor blood vessels, causes hemorrhage, and destroy the tumor [66, 67]. As a result of tumor vascular destruction of ROS by PDT in our study, the groups including PDT showed significant tumor volume reduction.

The Ki67, tumor cell proliferation and angiogenesis markers were investigated on the GBM (Fig. 8d and f, Immunofluorescence of Ki67). Ki-67 is expressed at a high frequency in high-grade brain tumors [68]. As a result, the Con and No laser groups in which a large volume of GBM was detected by Nissl and H&E staining also showed high level of Ki67 with mean fluorescence intensities (MFIs) of 9.7 ± 0.5 and 9.5 ± 0.5, respectively. However, in the PDT + PTT groups, the expression of Ki67 was remarkably reduced to 0.3 ± 0.4 and 0.8 ± 0.6 in both oral and IV administration, respectively. This is due to the synergistic effect of vasculature destruction and tumor cell apoptosis mediated by PDT and PTT.

Tumor destruction by phototherapy (PDT + PTT) results in immunogenic cell death (ICD), releasing damage-associated molecular patterns (DAMPs), and the immunogenicity of the tumor microenvironments increases [69, 70]. The release of DAMPs activates the immune system by inducing maturation of dendritic cells (DCs) that eventually migrate to the lymph node [71]. The high-mobility-group box 1 (HMGB1), an alarmin protein released from dying tumor cells, is considered a DAMP in cancer therapy. It acts as an endogenous ligand for toll-like receptor (TLR)-2, TLR-4, and TLR-9 [72]. Upon receptor binding, HMGB1 triggers the activation of signaling pathways and immune responses. However, based on the type of tissue, HMGB1 can either suppress tumor growth or promote oncogenesis. Therefore, the HMGB1 expression on the GBM after the treatments was investigated (Fig. 8d and g, Immunofluorescence of HMGB1). HMGB1 was expressed at high MFIs of 12.7 ± 0.5 and 12.8 ± 0.5 in the Con group and No laser group, respectively, which had a large amount of malignant GBM (Fig. 8e and g**).** On the other hand, the MFI gradually decreased in the experimental group treated with PDT or PTT, and further decreased in the combined PDT + PTT, to 0.7 ± 0.4 and 1.4 ± 0.4 in the oral and IV administration groups, respectively. A number of studies has found that higher expression of HMGB1 is closely associated with tumor angiogenesis, invasion, proliferation, and anti-apoptotic effects [73]. The released HMGB1 from GBM cells can activate AKT and ERK signaling pathways and promotes GBM cell invasion in this autocrine pathway [74]. Wang et al. [75] reported that a higher grade of glioma results in a higher level of HMGB1 expression. Furthermore, the expression of HMGB1 was examined in 15 samples of normal brain and 65 samples of various grades of glioma tissue (grades I to IV). HMGB1 was shown to be a promising prognostic biomarker for malignant glioma such as GBM. Consistent with recent studies of the high expression of Ki67 and HMGB1 in malignant GBM, those biomarkers were found with high frequency in the orthotopic GBM rat model presented in this study. However, their expressions was decreased by PDT and PTT treatments. This demonstrates that the PDT + PTT of Ce6-AuNP-Lf targeting GBM by oral administration or IV injection significantly destroy tumors.

### Inhibition of tumor growth by PDT + PTT of Ce6-AuNP-Lf in subcutaneous GBM mice model

The inhibition of tumor development by PDT + PTT of Ce6-AuNP-Lf was evaluated using C6-glioma subcutaneous mice model (Additional file 1: Figure S10a). On the 18th day after treatment, the tumor volume was 2874.2 ± 504.2 mm^3^, 1384.5 ± 37.8 mm^3^ and 1233.9 ± 196.1 mm^3^, in Con, IV and oral groups, respectively. The PDT + PTT of Ce6-AuNP-Lf suppressed tumor growth by 51.8 ± 1.3% and 57.1 ± 6.8% via IV and oral administration, respectively (Additional file 1: Figure S10b and S10c). In addition, there was no obvious decrease in body weight (Additional file 1: Figure S10d). TUNEL-positive apoptosis in the Ce6-AuNP-Lf treatment groups was remarkable in the laser irradiated area (Additional file 1: Figure S10e). The C6 glioma cell line is well known due to its high growth rate and vascularization and it is highly infiltrative [76]. However, the PDT + PTT of Ce6-AuNP-Lf administered by IV and oral effectively interfered with glioma progression and retarded the growth of a series of tumors. Consistent with the in vivo orthotopic GBM model, irreversible microvasculature destruction by PDT and apoptosis by PTT inhibit proliferation of the tumor. Angiogenesis is necessary for tumor progression. After the blood vessels that supply nutrients and oxygen to the tumor are blocked, then they become oligotrophic and hypoxic, which halts the process of carcinogenesis [77]. Therefore, the synergistic PDT + PTT of Ce6-AuNP-Lf can efficiently inhibit GBM growth and prolong survival by limiting angiogenesis, promoting tumor apoptosis, and inhibiting progression.

## Conclusions

Here we synthesized Ce6-AuNP-Lf, which exhibits potent PDT and PTT for GBM based on its physicochemical properties. The phenomenon that improves intrinsic ROS generation capacity of Ce6 os referred to as MERos, and it is mediated by intersystem crossover between Ce6 and AuNP. We also demonstrated that the ROS generation of Ce6-AuNP-Lf is maximized when Ce6 is intact and proximately conjugated to AuNP. Therefore, to improve PDT by MERos of the Ce6-AuNP-Lf, it should be applied before PTT, which offsets the therapeutic effect. Furthermore, Ce6-AuNP-Lf is capable of crossing both the intestinal barrier and BBB due to LfR-mediated transcytosis and the nano-size of Ce6-AuNP-Lf. Therefore, orally administered Ce6-AuNP-Lf has remarkable bioavailability and GBM accumulation. The MERos-mediated PDT and PTT of Ce6-AuNP-Lf resulted in significant tumor destruction and growth inhibition in various GBM mice models. Consequently, Ce6-AuNP-Lf is a good candidate as a phototherapeutic agent for GBM.

## Methods

### Materials

Gold (III) chloride trihydrate (HAuCl_4_), glutathione (GSH), human lactoferrin (hLf), sodium hydroxide (NaOH), thiourea solution (H_2_NCSNH_2_), sodium borohydride (NaBH_4_), 100-kDa and 10-kDa MWCO dialysis tubing cellulose membrane, 1-ethyl-3-(3-dimethylaminopropyl) carbodiimide (EDC), n-hydroxysuccinimide (NHS), osmium tetroxide (OsO_4_), pepsin, Spurr low-viscosity embedding kit, paraformaldehyde, cresyl violet-acetate, acetic acid, octanol, CHIR-99021, retinoic acid (RA), collagen IV, fibronectin, chlorpromazine hydrochloride and sodium acetate were purchased from Sigma-Aldrich, MO, USA. Chlorin e6 (Ce6, C_34_H_36_N_4_O_6_, MFCD08669566) was purchased from Frontier Scientific, USA. Centricon (MWCO; 3 kDa, UFC9003) was purchased from Millipore, Germany. Bifunctional poly(ethylene glycols) (SH-PEG-COOH) were purchased from Quanta BioDesign, Plain City, USA. JEM-301 HR-TEM grids were purchased from Nanolab Technologies, NY, USA. InstantBlue™ was purchased from Expedeon, UK. BCA protein assay kit was purchased from Pierce Biotechnology, Rockford, IL, USA. Transwell insert was purchased from Corning, Inc., Corning, New York, USA. Cell cytotoxicity assay EZ-Cytox was purchased from DoGenBio, Seoul, Korea. 3% isoflurane was purchased from HanaPharm, Seoul, Korea. A DeadEnd Fluorometric Terminal deoxynucleotidyl transferase dUTP nick end labeling (TUNEL) System kit was purchased from Promega, USA. Singlet oxygen sensor green (SOSG, S36002), 1 × B-27 Supplement, DMEM/F12, Knockout™ Serum Replacement, Non-Essential Amino Acids (100×), β-mercaptoethanol, human Endothelial SFM and GlutaMAX™ supplement were purchased from Thermo Fisher Scientific, USA. FITC-Dextran 3 kDa was purchased from Invitrogen, USA. Annexin V-DY-634/PI apoptosis stain (ab214484), anti-Ki67 antibody (ab15580), anti-HMGB1 antibody (ab18256), goat anti-rabbit IgG-H&L Alexa Fluor 488 (ab150077) and goat anti rabbit IgG H&L Alexa Fluor 647 (ab150079) were purchased from Abcam, UK. A DAPI mounting kit was purchased from Vector Laboratories, Inc., Burlingame, CA, USA. ELISA for IL6 (KET9007), IFN-γ (KET7017) and TNFα (KET9007) were purchased from Abbkine, Wuhan, China.

### Experimental cell lines and animals

Experiments were performed using the human umbilical vein endothelial cell line (HUVEC; LONZA, NJ, USA), the human epithelial colorectal cell line (Caco-2; Korean Cell Line Bank, Seoul, Korea), the C6 rat glioma cell line (Rockville, MD, USA), the U87MG human glioblastoma cell line (Korean Cell Line Bank, Seoul, Korea) and human brain microvascular endothelial cells (BMVECs) generated from human induced pluripotent stem cells (IMR90-4 iPSCs; WiCell Research Institute, Madison, WI, USA). HUVECs (passage numbers 4 to 6) were cultured using endothelial growth medium (EGM-2 bullet kit; LONZA, NJ, USA), and Caco-2, U87MG and C6 cells were cultured using Dulbecco’s modified Eagle’s medium (DMEM; GenDEPOT, TX, USA) containing 10% fetal bovine serum (FBS; GenDEPOT, TX, USA), and 1% penicillin–streptomycin in standard culture conditions at 37 °C and 5% CO_2._ The culture protocol for BMVECs derived from IMR90-4 iPSCs is described in the methodology section of human blood–brain barrier culture and formation of a cell monolayer.

In vivo experiments were carried out using five- to seven-week-old male Balb/c nude mice, Balb/c mice (Nara-Bio Company, Seoul, Korea), and seven- week- old male Sprague Dawley (SD) rats (DBL, Incheon, Korea). All animals were housed in specific pathogen-free conditions and maintained under the Institutional Animal Care and Use Committee (IACUC: 2020–0081) at Hanyang University.

### Preparation of glutathione-coated AuNPs (GSH-AuNPs)

A solution of HAuCl_4_ (11.1 mM), GSH (37.8 mM), and NaOH (178 mM) was dissolved in methanol/water (1.3:1 v/v, 20 mL). Next, this solution was diluted to a final Au^3+^ concentration (0.48 mM) with the addition of methanol (104 mL) and water (294 mL). The Au^3+^ was reduced with the addition of NaBH_4_ (0.25 M, 4 mL). The reduction of Au was allowed to proceed for 24 h at 100 °C with constant stirring. AuNP were precipitated with addition of NaCl (68 mM) in methanol (200 mL), followed by centrifugation (3200 rpm, 5 min). Precipitated AuNPs were reconstituted in DW. Their concentration was measured by UV–visible spectroscopy.

### Ce6 solution

Ce6 was diluted in a solution of sodium hydroxide (0.1 M) following the manufacturer’s instructions. Methanol was added to a final concentration of Ce6 (5 mM), and the pH was adjusted to 6.2. Fresh solutions were prepared for all experiments.

### Carbodiimide chemistry to functionalize Ce6 with thiourea

Ce6 solution (108 μL) was mixed with Sulfo-NHS (990 μL, 40.7 mg/mL) and EDC (900 μL, 16 mg/mL) in PBS (10 mM, pH 6.2). The solution was mixed every 5 min for 30 min. Then thiourea solution (3006 μL, 4 mM) was added and the solution was stirred occasionally for 120 min. Sodium hydroxide (42 μL, 0.1 M) was added to quench the reaction. The following thiourea-conjugated Ce6 (Ce6-thiol) purification process was used to remove excess unreacted reagents. Hydrochloric acid (3.3 μL) was added to the Ce6-thiol solution (1 mL). The sample was homogenized and centrifuged for 2 min at 15,000 rpm, the supernatant was discarded, and the pellet was resuspended with DW (600 μL). This process was repeated twice. Finally, the sample was resuspended with DW (200 μL) and stored at 4 °C.

### Surface anchoring of Ce6-thiol on the AuNPs

The Ce6-thiol solution (23 μM) was reacted with AuNP at a feed molar ratio of 1:1 and was stirred for 24 h. The molar extinction coefficient of Ce6 and AuNP is 45,000 cm^−1^ / M at 532 nm and 55,000 cm^−1^ / M at 671.0 nm, respectively [78]. The molarity of Ce6 and AuNP was calculated with UV–vis spectrometry according to the Beer Lambert law:

$$A\left(\lambda \right)={\mathrm{log}}_{10}\frac{{I}_{0}}{I}= \varepsilon c$$

where $A\left(\lambda \right)$ is the measured wavelength-dependent absorbance, ${I}_{0}$ is the incident light intensity, $I$ is the transmitted light intensity, $\varepsilon$ is the wavelength-dependent extinction coefficient of the substrate, $c$ is the concentration of the substrate and $l$ is the pathlength of the quartz cuvette (0.1 cm).

After 24 h, Centricon with a membrane pore size of 3 kDa NMWCO was used to eliminate the unconjugated-Ce6 and the resulting (Ce6-AuNPs) were reconstituted in DW (1 mL).

### Ce6-AuNP-Lf synthesis

Lactoferrin-conjugated poly (ethylene glycols) were synthesized by EDC/NHS amide bond linkage conjugation. Briefly, EDC (250 mM) and NHS (500 mM) were dissolved in PBS containing PEG (12.5 mM) with constant stirring. After approximately 15 min, PEG solution (12.5 mM) was added to PBS containing lactoferrin (0.125 mM) at 4 °C with constant stirring for 24 h. The resulting conjugates (Lf-PEG) were collected by dialysis, using a 10-kDa-pore membrane at 4 °C and were freeze-dried. A solution of Ce6-AuNP (70 μM) was mixed with Lf-PEG in PBS (5 mL) at 4 °C with constant stirring for 24 h. The resulting conjugates were collected by dialysis, using a 100-kDa-pore membrane at 4 °C, and were freeze-dried.

### Characterization of Ce6-AuNP-Lf

The specific peaks at 400 nm, 671 nm and 532 nm wavelengths of Ce6, AuNP, and Ce6-AuNP-Lf were observer using a UV–visible spectrophotometer (NanoDrop 2000; Thermo Scientific, Wilmington, DE, USA). Surface charge and dynamic light scattering were measured using a Zetasizer Nano ZS (Malvern, UK). The elemental mapping and sizes of the Ce6-AuNP and Ce6-AuNP-Lf were characterized by high-resolution TEM on a JEM-2100F (JEOL, Matsudo, Japan). HR-TEM grids were prepared by placing a drop of each sample on a carbon film-covered copper mesh grid for 1 min. The grids were allowed to air dry before being imaged by TEM. The heating profiles of Ce6-AuNP and Ce6-AuNP-Lf were measured using a thermal camera (FLIR C2, Wilsonville, OR, USA) and quantified by SigmaPlot 10.0 (System Software, CA, USA). A PTT laser (LRS-0532 DPSS Laser System, 532 nm; Laser glow Part Number: R5310B1FL, Toronto, ON, Canada) was applied for 240 s to vials containing Ce6-AuNP (10 µM of gold equivalent concentration) or Ce6-AuNP-Lf (10 µM of gold equivalent concentration).

### Hydrophobicity test of Ce6, Ce6-AuNP and Ce6-AuNP-Lf

Ce6, Ce6-AuNP, and Ce6-AuNP-Lf were dissolved in DW (600 μL), and their absorbance was measured at 400 nm and 671 nm. After that, octanol, which has strong hydrophobicity, was added to cause a phase transition. The hydrophobicity of drugs was determined by changes in absorbance.

### Photobleaching measurement of Ce6 fluorescence with laser irradiation

The fluorescence decays of Ce6, Ce6-AuNP, and Ce6-AuNP-Lf following laser irradiation were measured using fluorescence spectroscopy (Thermo Scientific™ VLBL00D0, USA). Each sample (2.5 μM of Ce6 equivalent concentration) was dissolved in ethanol and irradiated with a PDT laser (MRL-III-671, 671 ± 1 nm, China) for 10 min. The photobleaching of Ce6 fluorescence (λ excitation / λ emission = 400 nm / 671 nm) of samples was measured according to laser irradiation time.

### Singlet oxygen (^1^O_2_) generation capacity of Ce6-AuNP-Lf

PDT and PTT laser were applied to mixtures of SOSG reagent (0.5 μM) with Ce6, Ce6-AuNP, and Ce6-AuNP-Lf (2.5 μM of Ce6 equivalent concentration), respectively. Single laser groups (PTT or PDT) were irradiated for 5 min, and groups of a combination of lasers (PTT + PDT or PDT + PTT) were irradiated for 2.5 min for each laser. The generation of Singlet oxygen (^1^O_2_) was determined by measuring the fluorescence intensity (λ excitation / λ emission = 488 nm / 525 nm).

### Cytotoxicity evaluation of Ce6-AuNP-Lf

For cell viability assays, HUVECs and Caco-2 cells were seeded in 96-well plates at a seeding density of 5 × 10^3^ cells for each well and were incubated for 24 h in the CO_2_ incubator. Then, AuNP, Ce6-AuNP and Lf-PEG-AuNP (10 µM of gold equivalent concentration) were applied for 24 h. After washing with PBS buffer, wells were treated with culture medium containing EZ-Cytox at 37 °C and 5% CO_2_ for 4 h. The absorbance of the medium was measured with a micro-well plate reader at a wavelength of 450 nm.

### In vitro permeability capacity of Ce6-AuNP-Lf through Caco-2 cells

Caco-2 cells were seeded in a Transwell insert with 6.5-mm diameter and 0.4-μm pore size (seeding density: 2 × 10^4^ cells/insert; Corning, Inc., Corning, New York, USA). The cells were incubated for approximately 2–3 weeks in the CO_2_ incubator. To check the tight junction between them, the transepithelial electrical resistance (TEER) was measured using a voltmeter (EVOM2; World Precision Instruments, Sarasota, FL, USA). The TEER value > 3,700 Ω·cm^2^ was used for the assay. Caco-2 cells were treated with Ce6, Ce6-AuNP, or Ce6-AuNP-Lf (5 μM of gold and 2.5 μM of Ce6 equivalent concentration). For competitive binding, free Lf (5 μM) was pretreated for 2 h, followed by Ce6-AuNP-Lf (5 μM of gold equivalent concentration). During treatment, TEER values were measured at each designated time. To confirm the penetration of Ce6-AuNP and Ce6-AuNP-Lf through the Caco-2 cell monolayer, they were observed using TEM.

### Bio TEM epoxy resin fixation for detection of Ce6-AuNP and Ce6-AuNP-Lf

Sorensen’s phosphate buffer, which is composed of solutions A and B (A is 0.2 M Na_2_HPO_4_·2H_2_O and B is 0.2 M NaH_2_PO_4_·H_2_O), was added for 10 min to rinse off the fixative. Next, 1% osmium tetroxide (OsO_4_) was added to stain the Caco-2 cell monolayer or tissue membrane for 1 h. Then, the samples were washed with Sorensen’s phosphate buffer for 10 min to eliminate the remaining OsO_4_. Dehydration of the samples was performed with different concentrations of ethanol as follows: 30% for 10 min, 50% for 10 min, 70% for 10 min, 90% for 10 min, and finally, 100% for 20 min 3 times. The formation of epoxy resin block with a low-viscosity embedded media Spurr’s Kit method was applied to all the samples. The epoxy resin specimens were cut into 80 nm-thick sections using an ultramicrotome (EM UC7, Germany), and the obtained sections were air-dried for at least 1 h. Copper grids were mounted with 2% uranyl acetate for 20 min, briefly washed with distilled water, and mounted onto lead citrate (0.4%) for staining for 10 min. The section placed on the grid was observed using an 80 kV TEM.

### In vitro GBM cell uptake of Ce6-AuNP-Lf

U87MG cells (seeding density: 5 × 10^4^ cells/well) in a 4 well Nunc™ Lab-Tek™ II Chamber Slide™ System (Thermo Scientific™ 154526PK, USA) were treated with Ce6, Ce6-AuNP, or Ce6-AuNP-Lf (2.5 μM of Ce6 equivalent concentration) for 18 h. After washing three times with PBS, cells were fixed with 4% PFA for 15 min. Then, DAPI mounting medium (Vector Laboratories, Inc., Burlingame, CA, USA) was used and the intracellular Ce6 fluorescence was observed with a confocal microscope (TCS SP5, Leica, Germany). Flow cytometry (FACS Calibur™; BD Biosciences, Franklin Lakes, NJ) was used to quantify the intracellular Ce6 fluorescence. U87MG cells (80% confluency in 100 $\uppi$ culture dish) were treated with Ce6, Ce6-AuNP, or Ce6-AuNP-Lf (2.5 μM of Ce6 equivalent concentration) for 18 h. After washing three times with PBS, the cells were detached with trypsin/EDTA, centrifuged for 3 min at 1000 rpm, and analyzed with FACS.

### In vitro human blood–brain barrier culture and formation of cell monolayer

Human BMVECs were generated from human iPSCs as previously described [79] with modification of oxygen conditions [80] to mimic the hypoxic microenvironment of the developing brain. The human iPSCs line IMR90-4 (WiCell Research Institute) was maintained according to the WiCell Feeder Independent Pluripotent Stem Cell Protocol provided by the WiCell Research Institute. IMR90-4 iPSCs were singularized using Accutase™ and seeded on a 6-well plate coated with Corning Matrigel® at a density of 1.7 × 10^4^ cells per well in the presence of Y27632 (10 μM, Tocris Bioscience). Cells were cultured with TeSR™-E8™ (STEMCELL Technology) for three days until the cell density reached 3 × 10^5^ cells per well (D0-D3). To start differentiation into endothelial cells and neural progenitor cells, IMR90-4 iPSCs were switched from TeSR™-E8™ to unconditioned media (UM), which consisted of DMEM/F12 (78.5 mL), Knockout™ Serum Replacement (20 mL), Non-Essential Amino Acids (1 mL, 100 ×), GlutaMAX™ supplement (0.5 ml), and β-mercaptoethanol (182 μL) supplemented with CHIR-99021 (6 μM). On the next day, the cell culture media were switched to UM supplemented with 1 × B-27 Supplement without CHIR-99021 and they were changed daily for 5 days (D4-D9). For the next 2 days (D9-D11), the endothelial cells were selectively expanded by changing to endothelial cell media (EC); human Endothelial SFM supplemented with 20 ng/mL of basic fibroblast growth factor, 1 × B-27 Supplement, and retinoic acid (RA, 10 μM).

At day 11, cells were harvested from the 6-well plates using Accutase™ and seeded on the 0.4 μm-pore sized 24-well Transwell insert chamber coated with collagen IV (400 μg/ mL) and fibronectin (100 μg/mL) at a density of 3.3 × 10^4^ cells per insert. To recapitulate the phenotypic features of BBB, BMVECs seeded on the insert chamber were cultured with a mixture of human primary astrocytes (ScienCell) and pericytes (ScienCell) in the basal chamber.

At day 12, media were switched to EC without bFGF and RA and changed daily to maintain the BBB culture. From D6-D12, cells were cultured in a hypoxic incubator (EppendorfGalaxy® 48R) flushed with a 5% O_2_—5% CO_2_-N_2_ balance and transferred to a regular CO_2_ incubator.The impedance values for the BBB were measured using a TEER measurement machine (EVOM2, World Precision Instruments).

### Exposure of brain microvascular endothelial cell monolayers to NPs

The BBB penetration of Ce6-AuNP and Ce6-AuNP-Lf was evaluated in the Transwell-cultured human BBB model. For optimal results, human BBB models with TEER value > 3,700 Ω·cm^2^ were used for the assay and FITC-Dextran 3 kDa was used to monitor the barrier integrity of the BBB during the assay. The media were changed to Human Endothelial SFM media at 2 h before assay, and Ce6-AuNP and Ce6-AuNP-Lf (5 μM of gold equivalent concentration) were applied to the apical side of the in vitro BBB system in the absence of FITC-Dextran 3 kDa (250 μg/mL). The Transwell plates were incubated at 37 °C with agitation, and samples (200 μL) from the basal chamber were collected every 30 min for 2 h while adding the same volume of Human Endothelial SFM media to the basal chamber.

The quantification of NPs in the basal chamber was performed using an inductively coupled plasma mass spectrometer (ICP-MS, iCAP RQ; Thermo Fisher Scientific, USA) and the fluorescent intensities of the samples at excitation of 495 nm and emission of 519 nm were measured using a micro-plate reader (Thermo Scientific™ VLBL00D0, USA) to analyze the apparent permeability (P_app_). The P_app_ of NPs and FITC-Dextran was calculated as follows [81]:

$${P}_{app}=\frac{{V}_{b}\times {C}_{t}}{{C}_{0}\times \Delta t\times A}.$$

Here, *V*_*b*_ is the volume of basolateral chamber, *C*_*t*_ is the change in concentration, $\Delta {\varvec{t}}$ is change in time at steady state, A is the growth area (0.33 cm^**2**^ in the 24-well Transwell), and C_**0**_ is the initial concentration in the apical chamber.

To determine the endocytosis mechanism of Ce6-AuNP-Lf, the change in the P_app_ of Ce6-AuNP-Lf was determined in the presence of the clathrin-mediated endocytosis blocker, chlorpromazine hydrochloride. After 2 h of pre-treatment with chlorpromazine hydrochloride (50 μM), Ce6-AuNP-Lf (5 μM) was added to the apical chamber of the in vitro BBB system in the presence of inhibitor.

In addition, to identify the receptor specificity of Ce6-AuNP-Lf, Lf (5 μM) was pre-treated to the in vitro BBB system for 2 h. After 2 h of pre-treatment with Lf, Ce6-AuNP-Lf (5 μM) was added to the apical chamber of the in vitro BBB system in the presence of pre-treated Lf.

### *Epoxy resin fixation for Bio TEM of *in vitro* BBB model*

For Bio-TEM images of the Transwell-cultured human BBB model, the samples were fixed after 2 h exposure based on the experimental conditions for each group. Reagent setup and the procedure followed the reported method [82]. Next, 4% PFA solution was added to the apical and basolateral chambers of the Transwell filters (0.5 and 1.5 mL, respectively) for 1 h. Sorensen’s phosphate buffer, which is composed of solutions A and B (A is 0.2 M Na_2_HPO_4_·2H_2_O; B is 0.2 M NaH_2_PO4·H_2_O), was added for 10 min to rinse off the fixative. Next, 1% osmium tetroxide (OsO_4_) was added to stain the cell monolayer for 1 h. Sorensen’s phosphate buffer was used to wash the remaining OsO_4_ for 10 min. Dehydration of cell monolayers on both sides of the filters was performed with different concentrations of ethanol as follows: 30% for 10 min, 50% for 10 min, 70% for 10 min, 90% for 10 min, and finally 100% for 20 min three times. The formation of epoxy resin block with a Low Viscosity Embedded Media Spurr’s Kit method was applied to the entire Transwell containing the fixed cell monolayer. The epoxy resin block was cut to 4 mm, and the surrounding plastic and resin were removed by additional cutting to reveal the cubic alignment, including plastic, epoxy resin, filter membrane and epoxy resin from left to right. The specimens were cut into 80 nm-thick sections using a microtome and the obtained sections were air-dried for at least 1 h. Copper grids were mounted with 2% uranyl acetate for 20 min, briefly washed with DW and mounted onto lead citrate (0.4%) for staining for 10 min. The section placed on the grid was observed using an 80-kV transmission electron microscope.

### In vitro phototoxicity (PTT and PDT damage) of Ce6-AuNP-Lf in a GBM cell line

For phototoxicity measurements, U87MG cells (seeding density: 5 × 10^3^ cells/well) were treated with Ce6, Ce6-AuNP or Ce6-AuNP-Lf (5 μM of gold and 2.5 μM of Ce6 equivalent concentration) for 12 h. After washing three times with PBS, the cells were irradiated with a single laser (PTT or PDT) for 5 min or with a combination of lasers (PTT + PDT or PDT + PTT) for 2.5 min each. Cell viability was measured with an EZ-Cytox kit. The intracellular ROS generation by laser irradiation was measured using the fluorescent probe DCFH-DA following the manufacturer’s instructions. Annexin V-DY-634 / PI apoptosis was used to evaluate apoptosis. Briefly, cells were suspended in 400 μL binding buffer, followed by staining with Annexin V- DY-634 (5 μL) for 15 min at 2–8 °C. Next, PI (5 μL) was added and cells were incubated for 5 min. Cells were immediately analyzed using a FACS.

### In vivo pharmacokinetics (PK) study

For the pharmacokinetic study, Balb/c mice were dosed Ce6-AuNP-Lf at 30 mg/kg, 60 mg/kg, and 5 mg/kg via subcutaneous (SC), oral, and intravenous (IV), respectively. Blood samples (500 µl) were collected by intra-cardiac puncture at each time points of 10 min, 20 min, 30 min, 60 min, 2 h, 6 h, and 12 h after drug administration. The exact weights of blood samples were measured in a borosilicate glass tube. Next, 70% nitric acid (800 µl) was added to each glass tube and samples were heated at 60 °C in a hot water bath for 3 h. Thereafter, HCl (37%) was added to each glass tube, and samples were heated in the same conditions. The digested blood was transferred into 50 mL tubes and adjusted with 2% nitric acid and 0.5% HCl in DW. The solutions were filtered (0.22 µm pore-size) and analyzed by ICP-MS. The Au concentration was calculated and adjusted for sample weight. The standard curve of Au (0.0001 – 0.05 µg/mL) was linear, and the limit of detection was 0.0005 µg/mL. Background gold concentration, the measured pre-dose, was subtracted from measured values to derive the gold concentration attributed to the dose. Gold concentration in each sample was determined from the mean of six replicate measurements.

The bioavailability (BA) value was calculated as follows:

$$BA \left(F \%\right)= \frac{{AUC}_{ Oral or S.C}}{{AUC}_{ I.V}} \times \frac{{Dose}_{ I.V}}{{Dose}_{ Oral or S.C}}.$$

Here, *AUC *_*oral or SC*_ is the area under curve administered by oral or SC, respectively. *AUC *_*IV*_ is the area under curve administered by IV. *Dose *_*oral or SC*_ is the dosage of drug injected orally or via SC; *Dose *_*IV*_ is the dosage of drug injected via IV.

### In vivo fluorescence tracer image of Ce6-AuNP-Lf

For the fluorescence tracer image, Balb/c mice were dosed with Ce6-AuNP-Lf at 60 mg/kg. After 2, 6, 12, and 24 h, the experimental mice were sacrificed, and the organs were extracted. The fluorescence signals of Ce6-AuNP-Lf in organs were imaged using an in vivo imaging system (FOBI, CELLGENTEK, South Korea). The exposure time was fixed to 550 s for analyzing fluorescent signals from tissues.

### In vivo experiments using an orthotopic GBM mice model

To prepare the orthotopic GBM mice model, GBM cells (U87MG cell) were intracranially injected into 7-week-old male nude mice. Briefly, male nude mice were anesthetized with isoflurane (3%) and fixed by ear bar in a stereotaxic instrument (Stoelting Co., IL, USA). Once each mouse was anesthetized, the scalp at the surgical position was removed and a small hole positioned at 2 mm right lateral and 2 mm posterior from the bregma was drilled under sterile conditions. After that, PBS (containing 1 × 10^6^ U87MG cells, 8 μL) was loaded into a 26-G Hamilton syringe (Hamilton Company, NV, USA), and then the syringe was placed on the stereotaxic apparatus. After the needle of the syringe was positioned at a 3 mm depth, cells were injected with a 1 μL/min injection rate, followed by 3 min of waiting time to prevent overflow. After injection, the hole was sealed with bone wax and the scalp was closed with suturing. After this procedure, the mice were kept for 3 weeks until the injected cells reached the appropriate size of GBM tissue.

Previous histology studies have verified that GBM exhibits a spherical shape with a diameter of approximately 2 mm at the site of cell injection. Therefore, the boundary of GBM and normal brain was established from these shapes. To evaluate GBM targeting efficacy, Ce6-AuNP and Ce6-AuNP-Lf were administered via oral (0.07 µM of gold equivalent concentration) and IV (0.012 µM of gold equivalent concentration) methods, respectively. After 24 h of administration, GBM and normal brain were excised based on the boundary criterion and analyzed by ICP-MS and TEM.

To evaluate the phototherapeutic efficacy of Ce6-AuNP-Lf in an orthotopic GBM mice model, the groups were randomized into nine groups (n = 5): Con, oral No laser, IV No laser, oral PDT + PTT, oral PTT, oral PDT, IV PDT + PTT, IV PTT, and IV PDT. Then, Ce6-AuNP-Lf was administered via oral (0.07 µM of gold equivalent concentration) and IV (0.012 µM of gold equivalent concentration) methods, respectively. After 24 h of Ce6-AuNP-Lf administration, GBM was irradiated by single laser (PTT or PDT, 10 min) or by a combination of lasers (PDT + PTT, for 5 min each). The survival rate and body weight were monitored from day 1 to day 17 after beginning of treatment. On day 17, the brain in the mice was excised for histology analysis.

### In vivo experiments using orthotopic GBM rat model

Orthotopic GBM rat models were developed with seven-week-old male Sprague Dawley rats. The rats were anesthetized using 5% isoflurane in 70% N_2_O and 30% O_2_, and the skull was exposed to bore a 2-mm hole. The injection point was 2.0 mm lateral to bregma and was carefully drilled using a saline (0.89% NaCl) drip (coordinates to bregma: anteroposterior, 0 mm; lateral, 2.0 mm; ventral, 4.0 mm). C6 cells (1 × 10^5^ cells/10 μL) were injected into the cerebral cortex using a 26-gauge Hamilton syringe. Ten days after tumor implantation, the rats were randomly divided into 9 groups (n = 5): Con, oral No laser, IV No laser, oral PDT + PTT, oral PTT, oral PDT, IV PDT + PTT, IV PTT, and IV PDT. Ce6-AuNP-Lf was administered via oral (0.07 µM of gold equivalent concentration) and IV (0.012 µM of gold equivalent concentration) methods, respectively. After 24 h of Ce6-AuNP-Lf administration, GBM was irradiated by a single laser (PTT or PDT, 10 min) or by a combination of lasers (PDT + PTT, for 5 min each). On the day after laser irradiation, the blood was collected from the lateral saphenous vein using a 23-gauge needle. The collected blood was allowed to clot for 30 min at room temperature and then centrifuged for 10 min at 2,200 × g to obtain serum. The samples were divided into aliquots (1.0 mL) in a polypropylene tube and stored at -80 °C for further experiments. ELISAs for IL6, IFN-γ, and TNFα were performed with rat serum from each group according to the manufacturer’s manual. After three repeated treatment cycles, the rats were sacrificed by perfusion and the brains were harvested and fixed with 4% paraformaldehyde for further analysis.

### In vivo experiments using subcutaneous GBM xenograft mice model

Athymic Balb/c nude mice were inoculated subcutaneously in the right flank with PBS (containing 0.8 × 10^6^ C6 glioma cells, 100 μL). Five days after cell inoculation, the mice were randomly divided into 3 groups (n = 4). CON group received saline administration. Ce6-AuNP-Lf was administered via oral (0.07 µM of gold equivalent concentration) and IV (0.012 µM of gold equivalent concentration) methods, respectively. After 24 h, the PDT and PTT lasers were sequentially irradiated for 5 min. This treatment cycle was repeated three times and the survival rate, weight, and tumor size were monitored until day 18. Tumor size was determined using a caliper to measure the length **a** and width **b** of tumors and was calculated as $\frac{4}{3}\pi \times {a}^{2}\times {b}^{2}$, where a: smaller radius; b: larger radius. On day 18, the mice were sacrificed for further analysis.

### In vivo histological study for PDT and PTT efficacy of Ce6-AuNP-Lf on GBM in various GBM models

Tissues were immobilized in 4% paraformaldehyde for 2 days and then placed in a Leica TP1020 Semi-enclosed Benchtop Tissue Processor (Wetzlar, Germany) for washing, dehydration, clearing and paraffin infiltration of the tissue samples, followed by embedding in paraffin blocks. The blocks were transverse sectioned at a thickness of 6 μm with a Leica RM2145 Microtome (Wetzlar, Germany). For Nissl staining, the brain slides were stained with a stain solution prepared by dissolving cresyl violet-acetate (0.2 g) in distilled water (150 mL) and a buffer solution containing acetic acid (0.1 M) and sodium acetate (0.1 M). H&E staining and the TUNEL assay were used to detect the necrosis and apoptosis, respectively, in the GBM areas following the manufacturer’s instruction. For immunofluorescence (IF) staining, GBM tissue slides were stained with anti-Ki67 antibody and anti-HMGB1 antibody and, diluted 1:100 in phosphate buffered saline tween-20 (PBST) and goat serum mixture. Next, goat anti-rabbit IgG-H&L Alexa Fluor 488 and goat anti rabbit IgG H&L Alexa Fluor 647 were used as secondary antibodies, followed by DAPI mounting.

### Statistics

All data are expressed as mean ± standard error of the mean (SEM). Statistically significant differences were evaluated using Student’s *t*-tests or one-way analysis of variance (ANOVA) (Systat Software Inc., San Jose, CA, USA). *P*-values less than 0.05 were considered statistically significant.

## Supplementary Information

**Additional file 1: Figure S1.** Thiolation of Ce6 by amide bond formation. **Figure S2.** Hydrophobicity assay of Ce6, Ce6-AuNP and Ce6-AuNP-Lf. **Figure S3.** UV-vis absorbance of Ce6-AuNP-Lf under conditions that mimic the gastrointestinal microenvironment to which the substance is exposed when administered orally. **Figure S4.** UV-vis absorbance of Ce6-AuNP-Lf in (A) PBS and (B) 10% FBS over 180 days. **Figure S5.** Schematic illustration of the Pretreated-Lf/Ce6-AuNP-Lf group to verify only passive transport in the Caco-2 cell monolayer of Ce6-AuNP-Lf. **Figure S6.** Intracellular uptake of Ce6-AuNP-Lf. **Figure S7.** Annexin V-DY-634/PI apoptosis staining (ab214484, UK) for the apoptosis detection that occurred by irradiating lasers with nanoconjugates accumulated in U87MG. **Figure S8.** Confirmation of the specific GBM targeting of Ce6-AuNP-Lf through heat conversion property under PTT laser after 24 h oral administration (60 mg/kg). **Figure S9.** ROS generation of Ce6-AuNP and Ce6-AuNP-Lf at 1 cm thickness of live skin tissue irradiated with PDT laser (671 nm). **Figure S10.** Limiting tumor development by PDT+PTT of Ce6-AuNP-Lf in subcutaneous C6-glioma xenograft mice model.

**Additional file 2: Movie S1.** Temperature rises when PTT laser is irradiated to Ce6-AuNP-Lf.

**Additional file 3: Movie S2.** Temperature rises when PTT laser is irradiated to Ce6-AuNP.

## Data Availability

All data are available in the main text or the additional materials.

## Abbreviations

Ce6: Chlorin e6
Lf-PEG: COOH-PEG-SH conjugated to lactoferrin
NP: Nanoparticles
AuNP: Gold nanoparticles
GSH: Glutathione
Ce6-AuNP: Ce6 conjugated AuNP
Ce6-AuNP-Lf: Lf-PEG conjugated to Ce6-AuNP surface
Lf: Lactoferrin
LfR: Lactoferrin receptor
PDT: Photodynamic therapy
PTT: Photothermal therapy
NIR: Near infrared
MEF: Metal enhanced fluorescence
MERos: Metal enhanced ROS generation
DW: Distilled water
SPR: Surface plasmon resonance
GBM: Glioblastoma multiforme
BBB: Blood–brain barrier
GI tract: Gastrointestinal tract
EPR: Enhanced permeability and retention
TEER: Transepithelial electrical resistance
ICP-MS: Inductively coupled plasma-mass spectroscopy
Bio TEM: Bio-transmission electron microscopic
TUNEL: Terminal deoxynucleotidyl transferase dUTP nick end labeling
SEM: Standard error of the mean
AUC: Area under the curve
