# Supplementary material for: A novel therapeutic strategy of multimodal nanoconjugates for state-of-the-art brain tumor phototherapy
Source: J Nanobiotechnology. 2022 Jan 4;20:14. doi: 10.1186/s12951-021-01220-9 (PMC8725459; doi:10.1186/s12951-021-01220-9)
Supplement: Supplementary file 1 — Additional file 1: Figure S1. Thiolation of Ce6 by amide bond formation. Figure S2. Hydrophobicity assay of Ce6, Ce6-AuNP and Ce6-AuNP-Lf. Figure S3. UV-vis absorbance of Ce6-AuNP-Lf under conditions that mimic the gastrointestinal microenvironment to which the substance is exposed when administered orally. Figure S4. UV-vis absorbance of Ce6-AuNP-Lf in (A) PBS and (B) 10% FBS over 180 days. Figure S5. Schematic illustration of the Pretreated-Lf/Ce6-AuNP-Lf group to verify only passive transport in the Caco-2 cell monolayer of Ce6-AuNP-Lf. Figure S6. Intracellular uptake of Ce6-AuNP-Lf. Figure S7. Annexin V-DY-634/PI apoptosis staining (ab214484, UK) for the apoptosis detection that occurred by irradiating lasers with nanoconjugates accumulated in U87MG. Figure S8. Confirmation of the specific GBM targeting of Ce6-AuNP-Lf through heat conversion property under PTT laser after 24 h oral administration (60 mg/kg). Figure S9. ROS generation of Ce6-AuNP and Ce6-AuNP-Lf at 1 cm thickness of live skin tissue irradiated with PDT laser (671 nm). Figure S10. Limiting tumor development by PDT+PTT of Ce6-AuNP-Lf in subcutaneous C6-glioma xenograft mice model. [file 12951_2021_1220_MOESM1_ESM.docx]

**Supplementary Information for**

**A novel therapeutic strategy of multimodal nanoconjugates for state-of-the-art brain tumor phototherapy**

Hyung Shik Kim^1^, Minwook Seo^2^, Tae-Eun Park^2^, and Dong Yun Lee^1,3,4,*^

***Correspondence Author:** Dong Yun Lee, Ph.D., e-mail: [dongyunlee@hanyang.ac.kr](mailto:dongyunlee@hanyang.ac.kr)

**Contents:**

Supplementary Figure S1~S10

Supplementary Movie S1 and S2

**ABBREVIATIONS**

Ce6, Chlorin e6; Lf-PEG, COOH-PEG-SH conjugated to lactoferrin; NP, nanoparticles; AuNP, gold nanoparticles; GSH, glutathione; Ce6-AuNP, Ce6 conjugated AuNP; Ce6-AuNP-Lf, Lf-PEG conjugated to Ce6-AuNP surface; Lf, lactoferrin; LfR, lactoferrin receptor; PDT, photodynamic therapy; PTT, photothermal therapy; NIR, near infrared; MEF, metal enhanced fluorescence; MERos, metal enhanced ROS generation; DW, distilled water; SPR, surface plasmon resonance; GBM, glioblastoma multiforme; BBB, blood-brain barrier; GI tract, gastrointestinal tract; EPR, enhanced permeability and retention; TEER, transepithelial electrical resistance; ICP-MS, inductively coupled plasma-mass spectroscopy; Bio TEM, bio-transmission electron microscopic; TUNEL, terminal deoxynucleotidyl transferase dUTP nick end labeling; SEM, standard error of the mean; AUC, area under the curve.


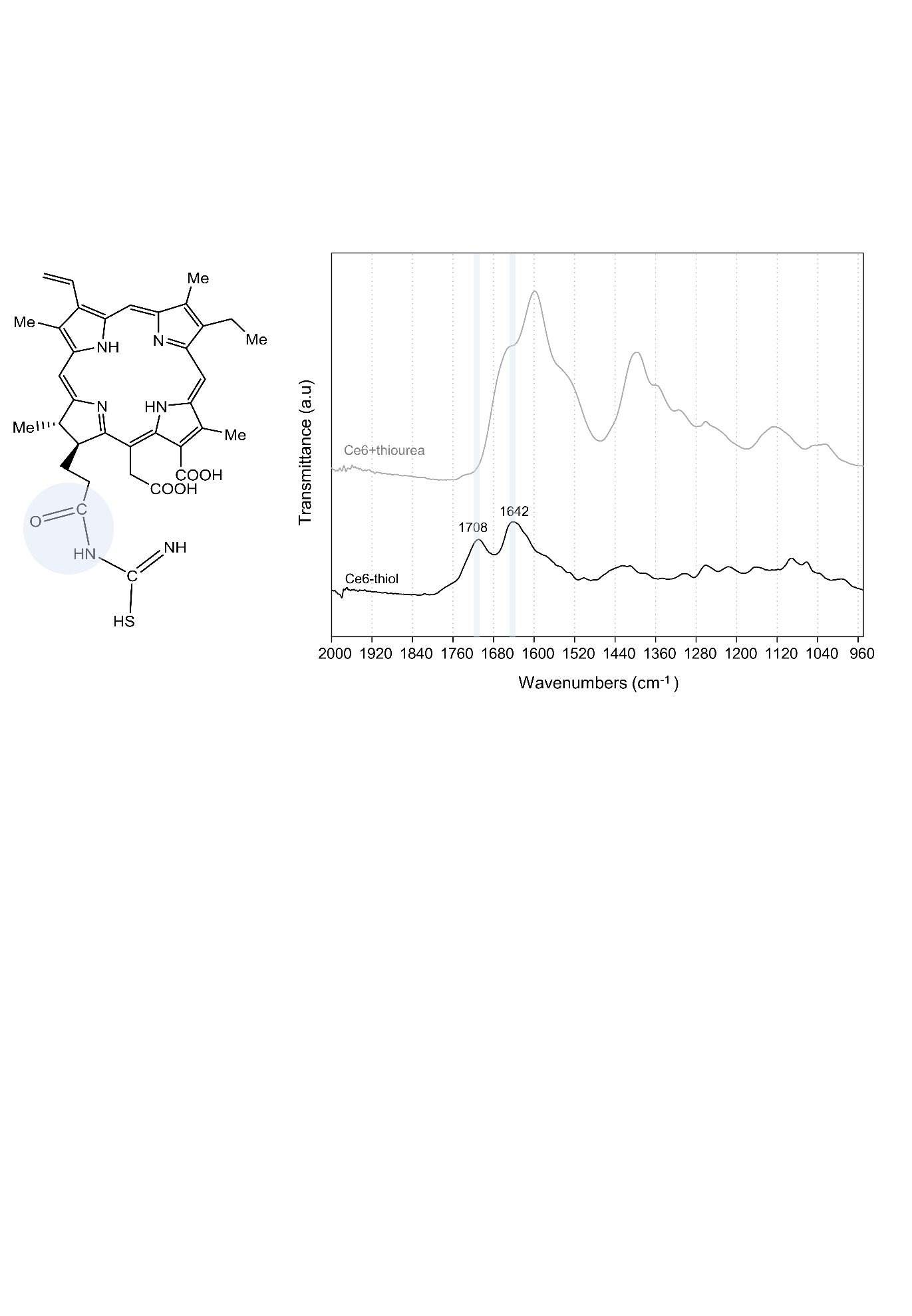


**Figure S1. Thiolation of Ce6 by amide bond formation.** FT-IR absorption spectra bands at 1708 cm-1, which corresponds to the C=O stretching of carboxylic acids in Ce6 , a band at 1642 cm-1 in the vibrational mode of the amide I bond.


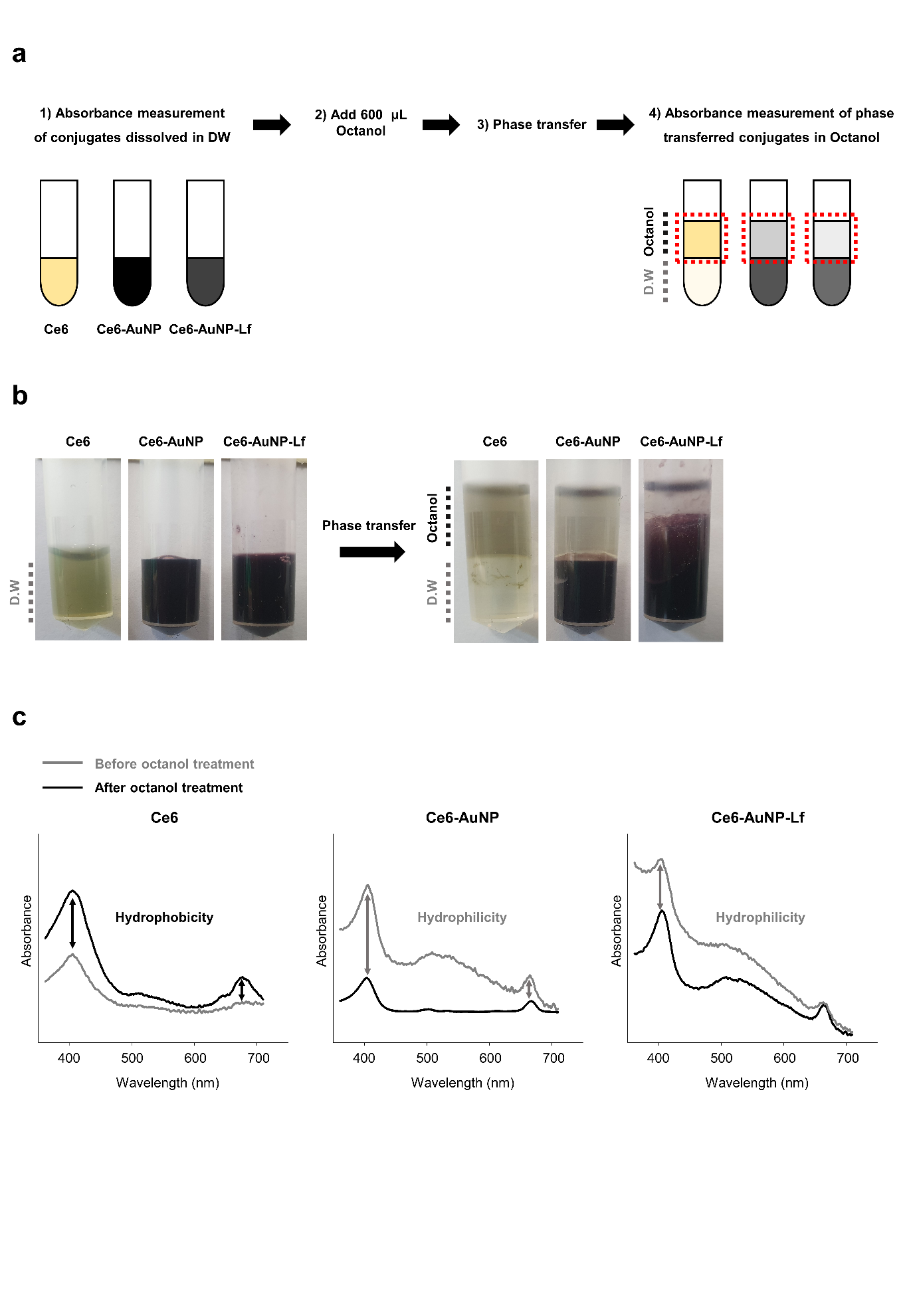


**Figure S2. Hydrophobicity assay of Ce6, Ce6-AuNP and Ce6-AuNP-Lf.** (a) Schematic illustration of measuring the hydrophobicity of Ce6, Ce6-AuNP and Ce6-AuNP-Lf, respectively. (b) Phase transferring between Octanol and DW according to the respective hydrophobicity of Ce6, Ce6-AuNP and Ce6-AuNP-Lf. (c) The absorbance changed by phase transfer caused by the addition of Octanol.


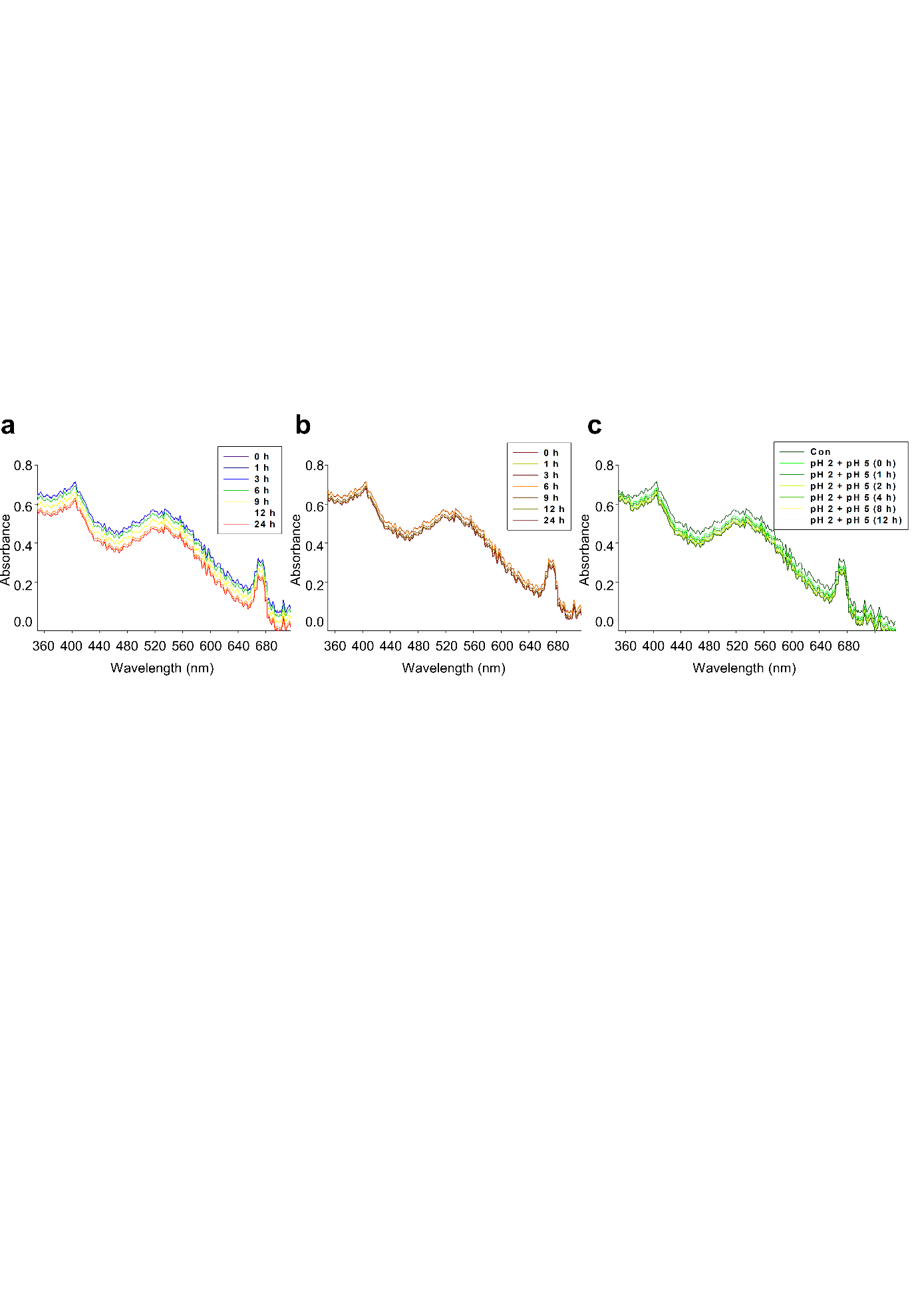


**Figure S3. UV-vis absorbance of Ce6-AuNP-Lf under conditions that mimic the gastrointestinal microenvironment to which the substance is exposed when administered orally.** (a) pH 2, (b) pH 5 over 24 h, (c) pH 2 for 3 h and transfer to pH 5 over additional 12 h. AuNP absorbance peaks at 532 nm and Ce6 absorbance peak at 671 nm without any shift over time. At pH 2, the absorbance value slightly decreased after 6 h, but under the conditions most accurately mimicking the gastrointestinal microenvironment, the change in absorbance was insignificant for a total of 15 h.


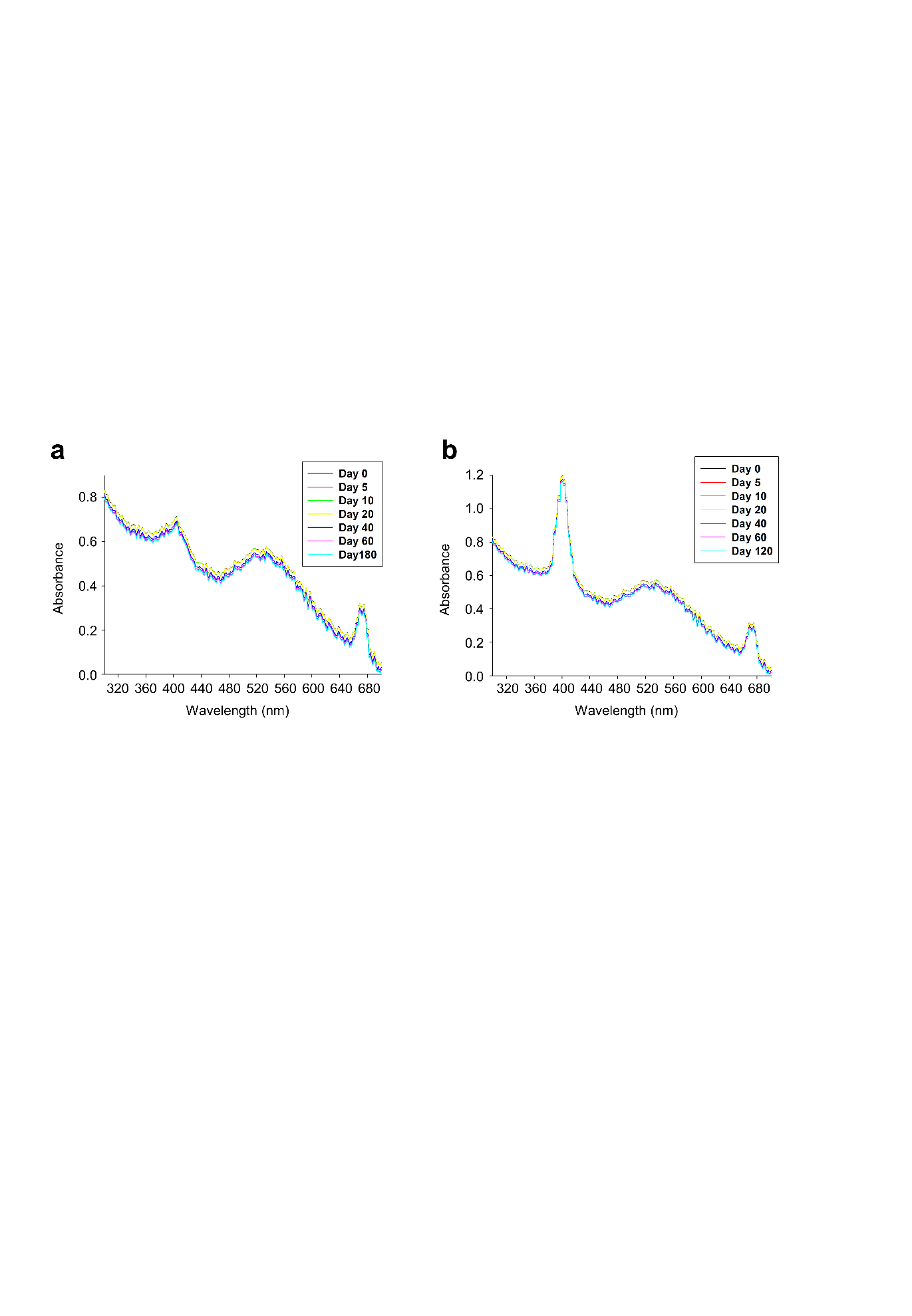


**Figure S4. UV-vis absorbance of Ce6-AuNP-Lf in (A) PBS and (B) 10% FBS over 180 days.** The peak at 400 nm (b) is due to the absorbance of bovine hemoglobin (BHb) in FBS while the AuNP absorbance peaks at 532 nm without any shift over 180 days. The peak at 671 nm originates from the conjugated Ce6 at the AuNPs surface and its absorbance intensity did not change, indicating that tight anchoring of Ce6 on the AuNPs without any denaturation for prolonged time.


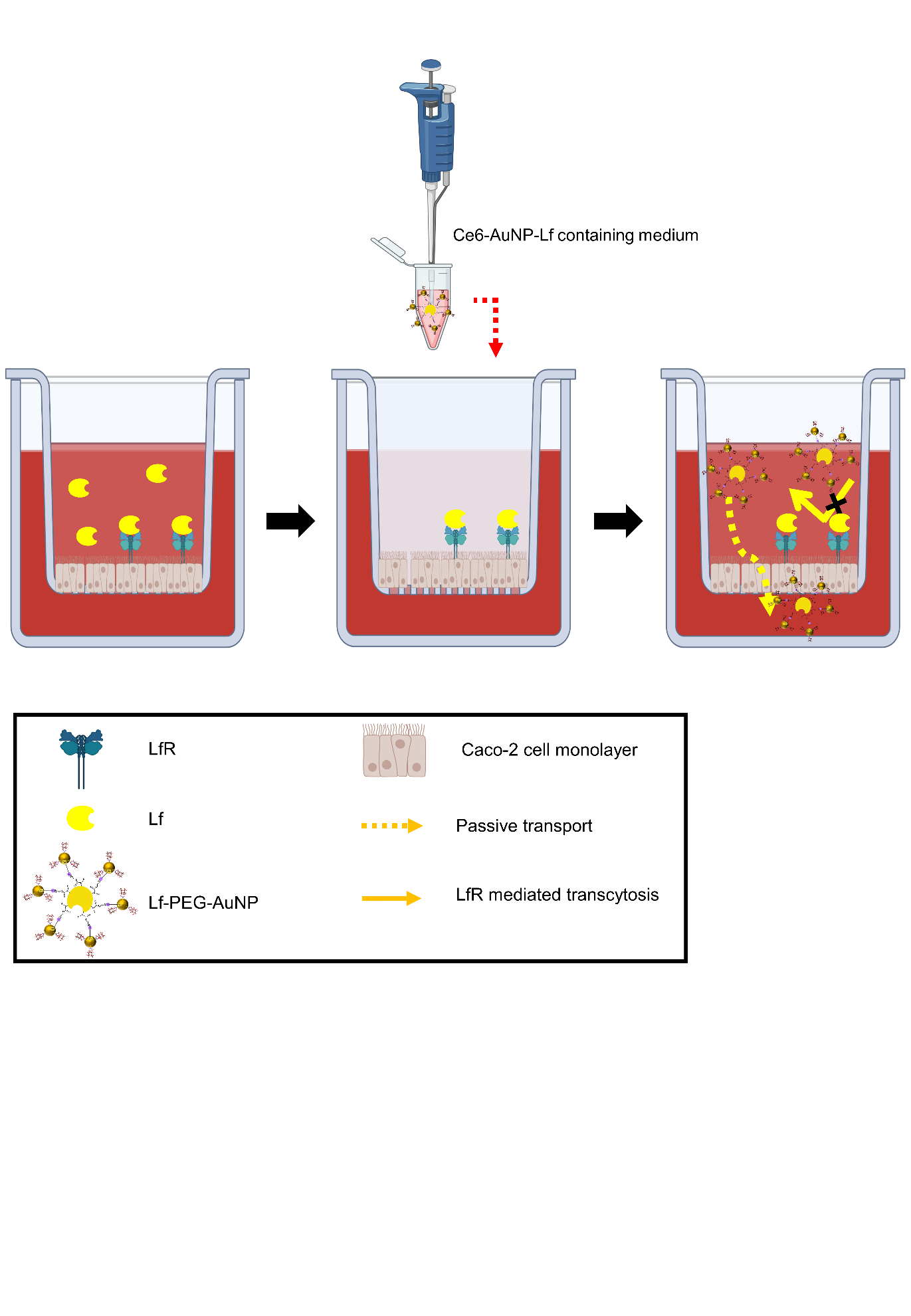


**Figure S5. Schematic illustration of the Pretreated-Lf / Ce6-AuNP-Lf group to verify only passive transport in the Caco-2 cell monolayer of Ce6-AuNP-Lf.** Pretreatment of Lf to saturate LfR that expressed on the Caco-2 cell monolayer. After 2 h of Lf pretreatment, the medium is replaced with Ce6-AuNP-Lf containing medium. Thereafter, Ce6-AuNP-Lf not able to transcytosis through LfR because of pretreated Lf, and only penetrate Caco-2 cell monolayer through passive transport between tight junctions. The illustration was created with BioRender.com.


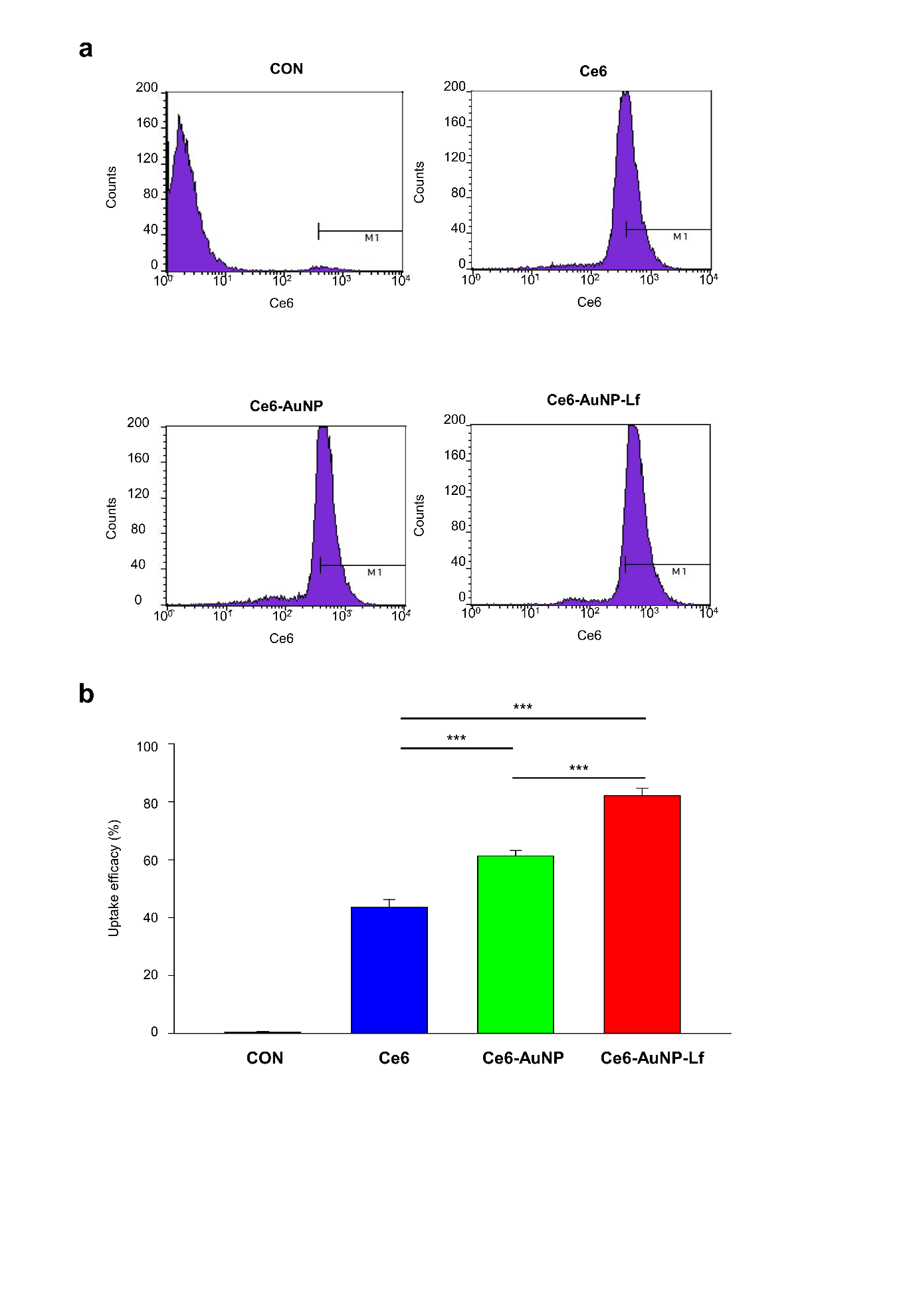


**Figure S6.** (a) Histogram data of flow cytometry (FACS Calibur^TM^; BD Biosciences, Franklin Lakes, NJ), (b) Uptake efficacy with results of the intracellular Ce6 fluorescence of the each nanoconjugates; Ce6-AuNP-Lf, Ce6-AuNP and Ce6 that treated 18 h on the U87MG with 2.5 µM of Ce6 equivalent concentration. Data were expressed as the mean ± S.E.M (n=3). ***P < 0.001


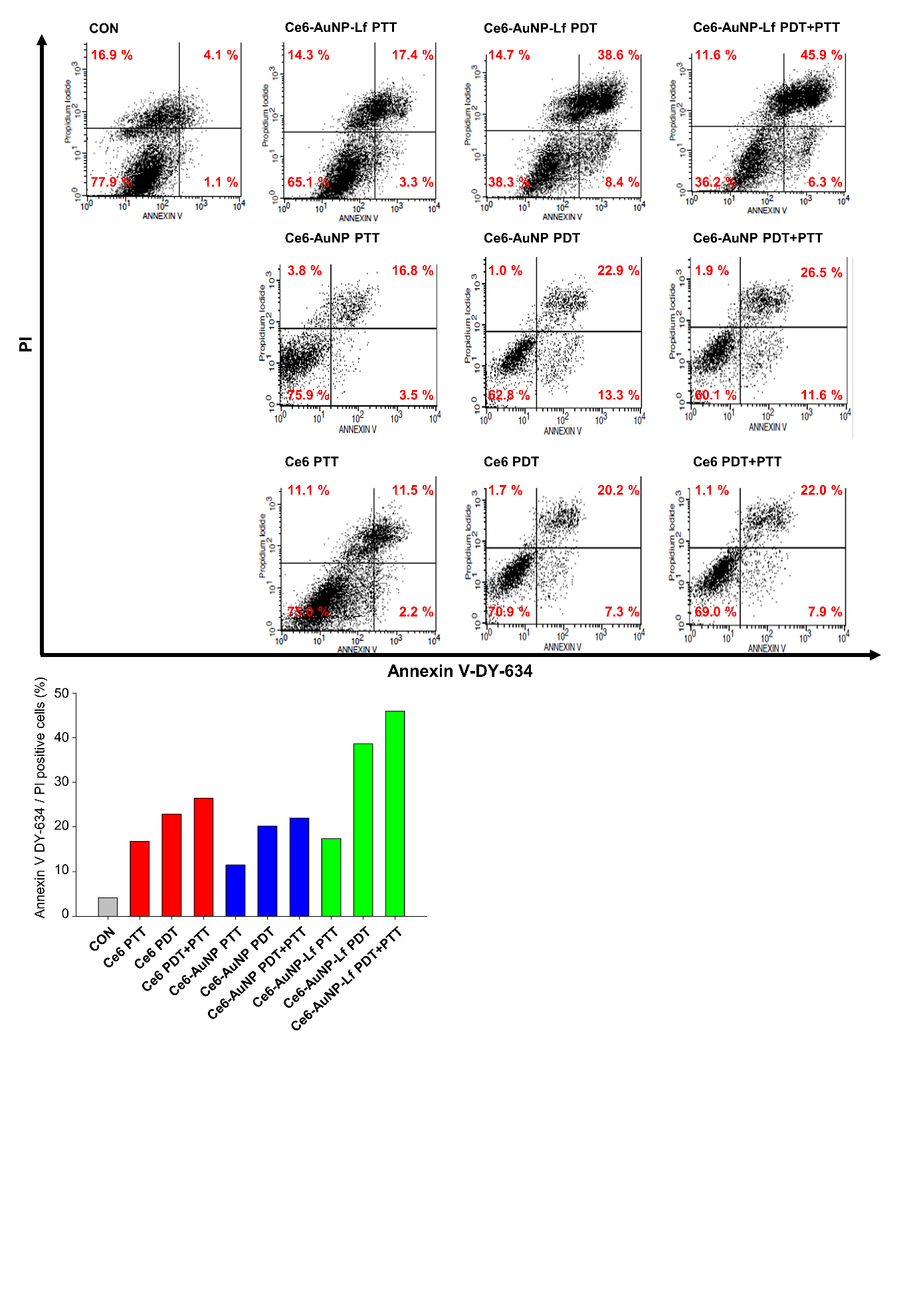


**Figure S7. Annexin V-DY-634 / PI apoptosis staining (ab214484, UK) for the apoptosis detection that occurred by irradiating lasers with nanoconjugates accumulated in U87MG.** U87MG cells at a density of 5 × 10^3^ cells/well were treated with media containing equivalent concentration of 2.5 µM and 10 µM of Ce6 and AuNP respectively for 12 h. After several washing with PBS, the cells were irradiated using PTT and PDT lasers under the conditions set for each experimental group. Then, the irradiated cells were suspended in 400 μL binding buffer, followed by staining with 5 μL of Annexin V- DY-634 for 15 min at 2–8 °C, and then 5 μL of propidium iodide (PI) was added and incubated for 5 min. Cells were immediately analyzed by FACS Calibur^TM^.


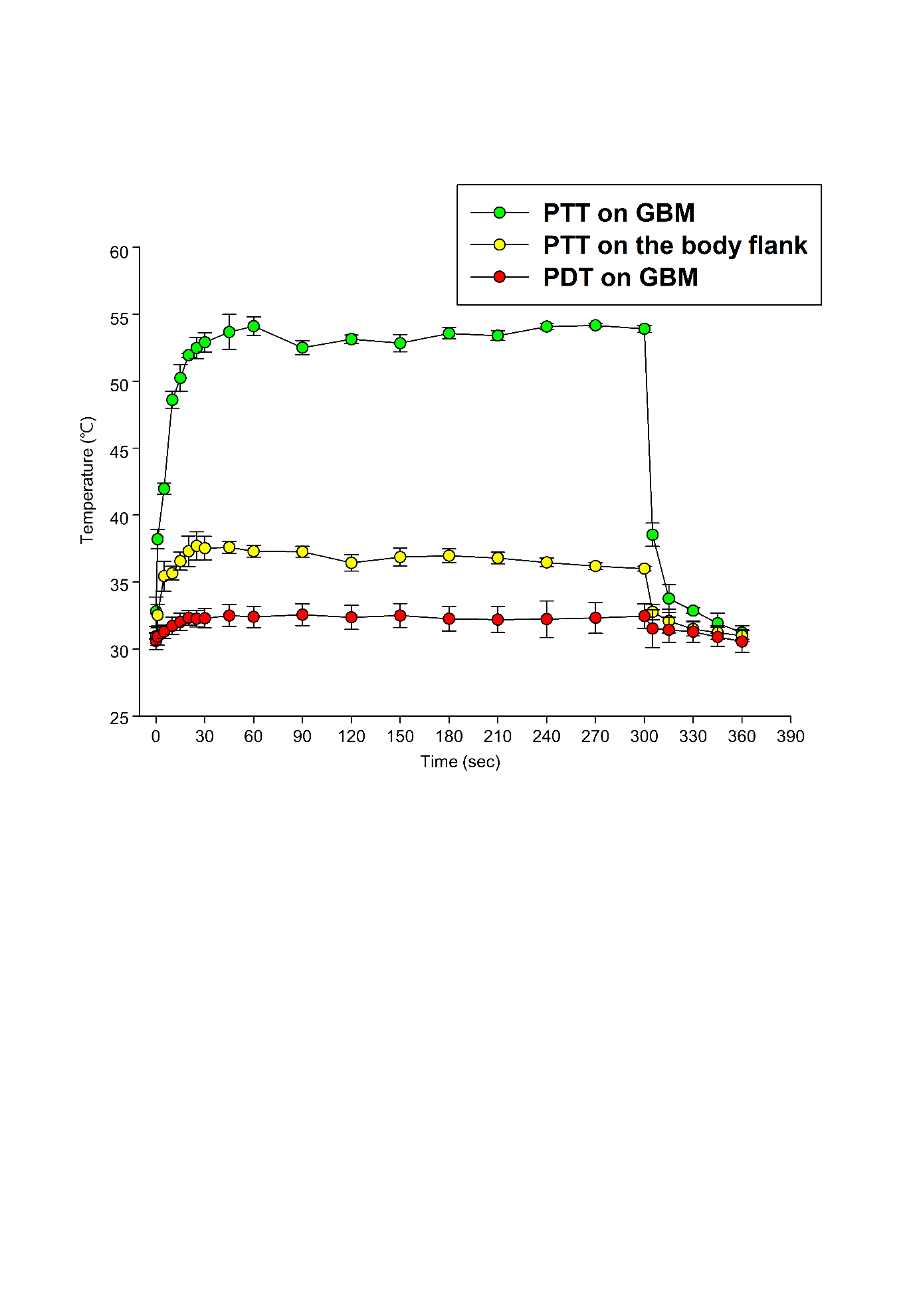


**Figure S8**. **Confirmation of the specific GBM targeting of Ce6-AuNP-Lf through heat conversion property under PTT laser after 24 h oral administration (60 mg/kg).** The heating profiles were confirmed using a thermal camera (FLIR C2, Oregon, Wilsonville, USA). PTT laser (LRS-0532 DPSS Laser System, 532 nm; Laser glow Part Number: R5310B1FL, Toronto, ON, Canada) was applied to GBM region and the body flank for 300 sec and 60 sec off state. PDT laser (LRS-0671 DPSS Laser System, 671 nm; Laser glow Part Number: R5310B1FL, Toronto, ON, Canada) also applied to GBM region for same time interval; Data were expressed as mean ± S.E.M (n=5).


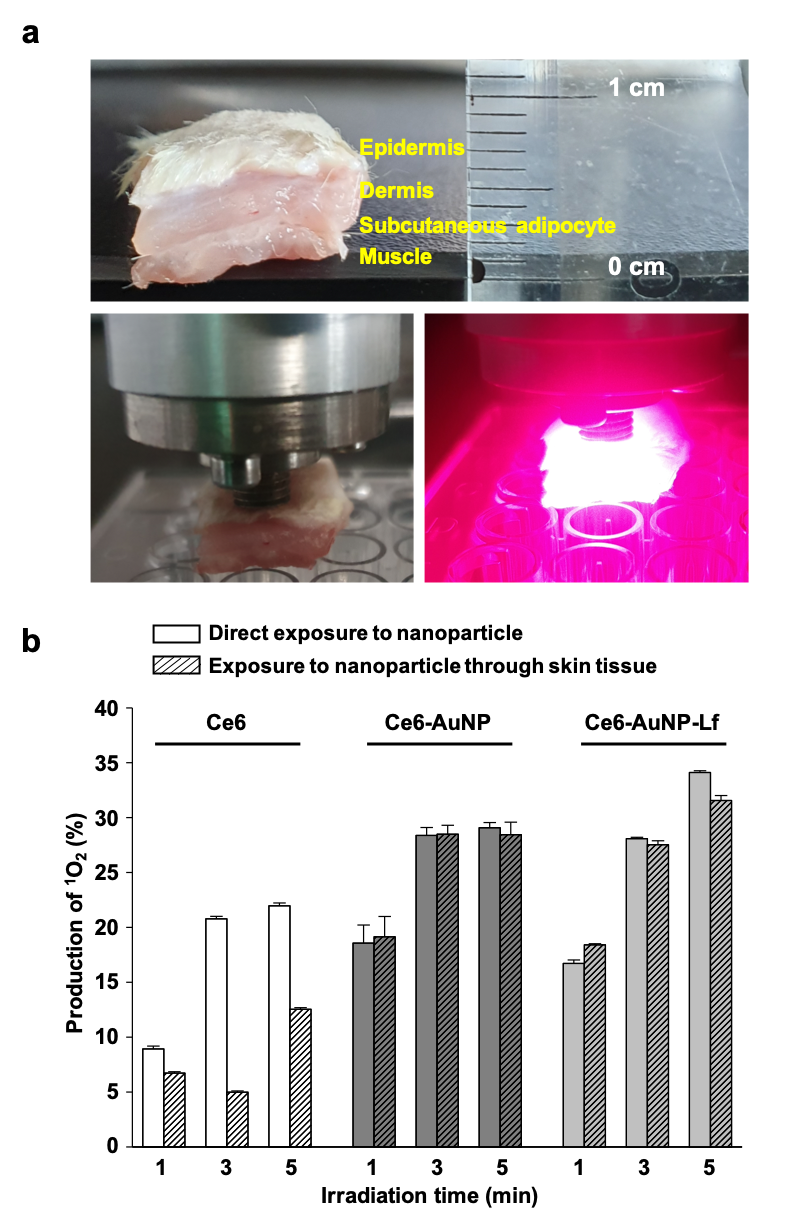


**Figure S9. ROS generation of Ce6-AuNP and Ce6-AuNP-Lf at 1 cm thickness of live skin tissue irradiated with PDT laser (671 nm).** (a) Live skin tissue irradiated with PDT laser (671 nm). 1 cm thick biological tissue was extracted from the skin of mice. 10 μL of DPBF solution (10 mg/mL in DMSO) was added to 1.5 mL water-diluted Ce6 and Ce6-AuNP, Ce6-AuNP-Lf (10 μM of Ce6 equivalent concentration). Then the NIR laser (671 nm) was irradiated for 1, 3, 5 min with or without the biological tissue interposed. (b) Production of ^1^O_2._ The absorption values at 424 nm before (A0) and after (A1) irradiation were recorded and normalized (100 X (A0 - A1)/A0) to quantify the production of ^1^O_2._ Data are expressed as mean ± SEM (n=3).


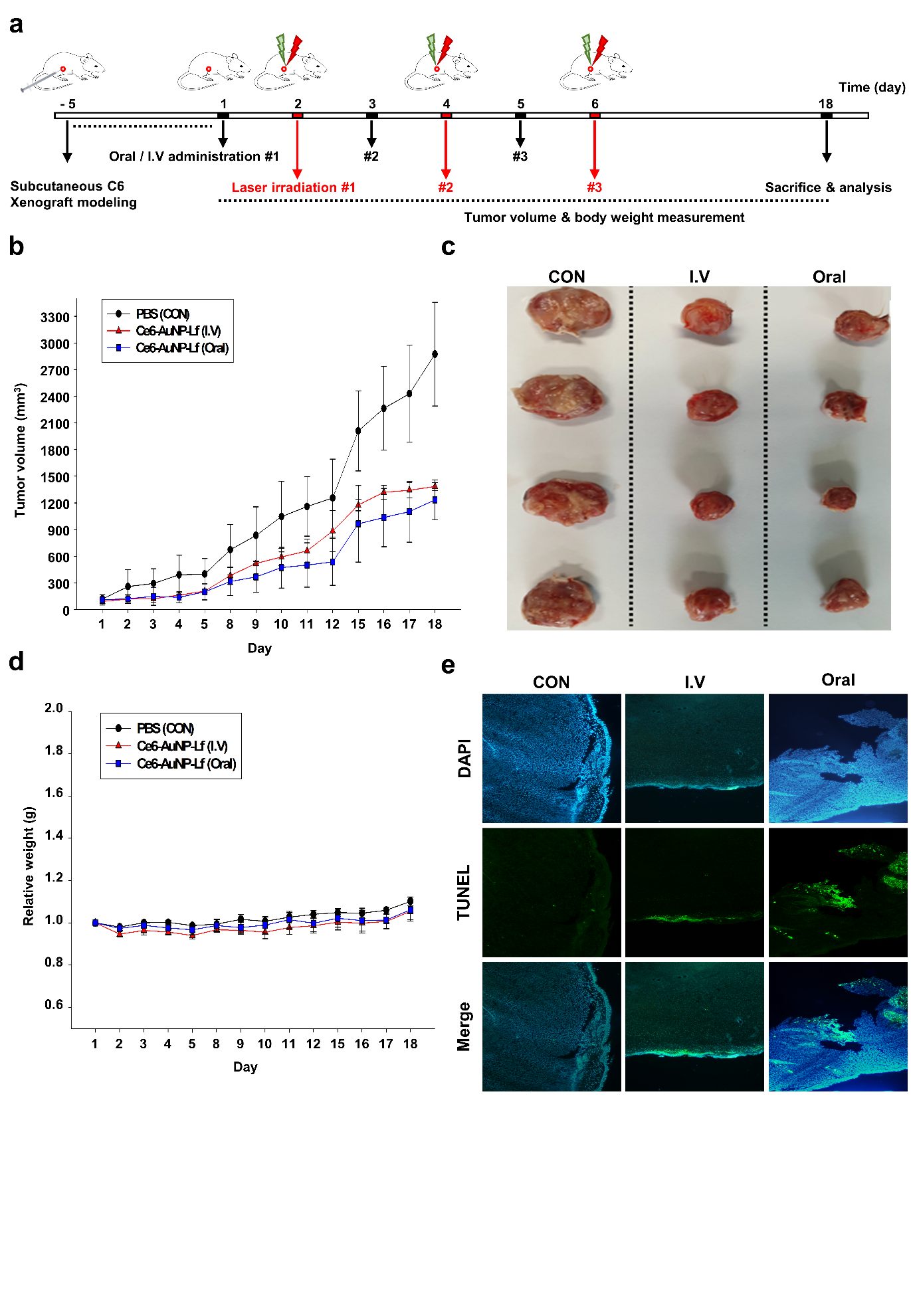


**Figure S10. Limiting tumor development by PDT+PTT of Ce6-AuNP-Lf in subcutaneous C6-glioma xenograft mice model.** (a) Schematic illustration of treatment cycle including drug administration, laser irradiation and monitoring the survival rate and body weight transformation. (b) Tumor volume of each groups. Data were expressed as mean ± S.E.M (n=4). (c) Representative tumor image of mice models after day 23. (d) Relative body weight of each groups, that monitored for 18 day. Data were expressed as mean ± S.E.M (n=4). (e) TUNEL-positive apoptosis in the tumor.

**Supplementary Movie S1**. Temperature rises when PTT laser is irradiated to Ce6-AuNP-Lf.

**Supplementary Movie S2**. Temperature rises when PTT laser is irradiated to Ce6-AuNP.
